# Mechanism of Pyrolysis Reaction of Al-Rich Al/PTFE/TiH_2_ Active Material

**DOI:** 10.3390/polym13172857

**Published:** 2021-08-25

**Authors:** Yilei Wang, Chunlan Jiang, Yuande Fang, Xinyu Wang, Zaicheng Wang

**Affiliations:** State Key Laboratory of Explosion Science and Technology, Beijing Institute of Technology, Beijing 100081, China; wylbitDr@163.com (Y.W.); 3120190182@bit.edu.cn (Y.F.); wangkingodwin@163.com (X.W.)

**Keywords:** Al-rich Al/PTFE/TiH_2_ composite, wet mixing, thermogravimetry differential scanning calorimetry, X-ray diffractometer, oxygen bomb calorimeter, component analysis

## Abstract

In order to obtain the chemical reaction mechanism of Al-rich Al/PTFE/TiH_2_ composites in argon and oxygen atmosphere, Al/PTFE, PTFE/TiH_2_, Al/TiH_2_ and Al-rich Al/PTFE/TiH_2_ with different contents of TiH_2_ composites were prepared by using the wet mixing method. The pyrolysis behavior of the above composites was investigated by thermogravimetric differential scanning calorimeter (TG-DSC). In addition, the calorific value of the above composite was measured by an oxygen bomb calorimeter. The compositions of TG-DSC residues at different peak temperatures and 1000 °C and the residues of oxygen bomb experiment were analyzed by X-ray diffraction (XRD), The results show that the pyrolytic products of Al-rich Al/PTFE/TiH_2_ materials under argon atmosphere can be divided into four stages. In the first stage (328.6–378.6 °C), the products are TiH_1.924_, (C_2_F_4_)_n_, (CF_2_)_n_, H_2_(g), Al and TiH_2_; in the second stage (510.8–534.3 °C), the products are Al, TiH_1.924_, (C_2_F_4_)_n_, (CF_2_)_n_, Ti, AlF_3_, TiF_3_, TiF_4_(g), C and H_2_(g). In the third stage (540.8–618.1 °C), the products are Al, C, Ti, (C_2_F_4_)_n_, (CF_2_)_n_, AlF_3_, TiF_3_, TiF_4_, CF_4_(g), C_3_F_6_(g), C_4_F_8_(g), C_2_F_6_(g), Al_5_Ti_2_ and H_2_(g); in the fourth stage (918.5–1000 °C), the products are AlCTi_2_, Al_2_Ti, AlTi, TiC, AlF_3_, Al, TiF_3_, TiC_0.957_, TiC_0.981_ and TiC_0.95_. The calorific value of the combustion of Al-rich Al/PTFE/TiH_2_ composite with 10% the content of TiH_2_ is the highest and is 19,899 J/g, which is 3.776% higher than that of Al-rich Al/PTFE composite. When TiH_2_ content is greater than zero and not more than 10%, the chemical reaction mechanism of Al-rich Al/PTFE/TiH_2_ is almost the same under oxygen atmosphere. When the content of TiH_2_ is higher than 10%, the mechanism of this material is different.

## 1. Introduction

Active material, also known as reactive material, is a typical impact initiation energetic material [1,2,3], which is composed of at least two kinds of nonexplosive materials. Due to the unique mechanical and chemical properties of the active materials [4,5,6,7,8,9,10], it has high application values in military fields such as air defense, antimissile barrier breaking and so on. Distinct from the traditional inert damage elements such as tungsten and steel alloy, which rely on a single kinetic energy to hit the target, the damage element made of active materials produces high temperature combustion and explosion effects when hitting the target at high speed and releases a large amount of chemical energy quickly, causing more damage to the target. By comparing the damage effects of the warhead with the warhead with active and inert damage elements, the U.S. Naval Research Office found that the kill radius of the warhead with an active damage element is twice of that of the warhead with the inert damage element; the damage power is greater by five times compared to the inert damage element warhead; the chemical energy of the active damage element is about 12-times of the average kinetic energy of the inert damage element [11]. In recent years, scholars at home and abroad have carried out a large number of experimental studies based on Al/PTFE active materials [12,13,14,15]. Ames [16,17], Wang [18], Zhou [19] and others carried out research on the energy release characteristics of active materials with the help of quasi-closed reaction vessels. Ren [20] studied the preparation, mechanical properties and impact sensitivity of PTFE/Al/MnO_2_ composites. Zhao [21] used SHPB experimental technology in order to study the mechanical properties and loading reaction properties of PTFE/Al with two ratios at high strain rate. The effects of the difference of aluminum content on the yield strength, failure property and reaction property of PTFE/Al active materials were compared and analyzed. 

**Figure 1:**
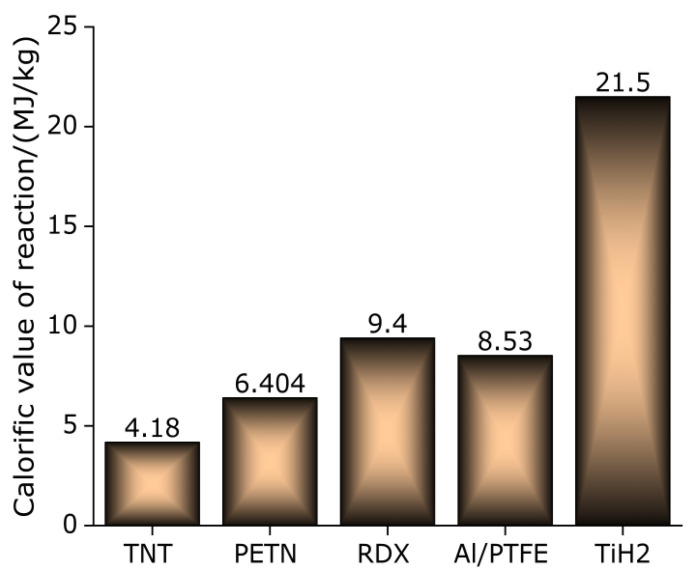
Plane bar chart of reaction calorific value of various materials.

Metal hydride has excellent hydrogen storage performance and higher energy density. As an example of high energy additive titanium hydride (TiH_2_), it has the following advantages:

1. TiH_2_ begins to decompose and release hydrogen (H_2_) when heated to 400 °C under the protection of Ar [22,23], and it is completely dehydrogenated at 600–800 °C [24].
2. It has high concentrations of hydrogen. The highest hydrogen storage rate can reach 4%.
3. The calorific value of the reaction is high. When the hydrogen content is 3.9%, the calorific value of combustion of titanium hydride is 21.5 MJ/kg [25]. Figure 1 shows the reaction calorific values of TiH_2_, Al / PTFE and three kinds of high explosives (Trinitrotoluene (TNT), Pentaerythritol tetranitrate (PETN), Cyclotrimethylene trinitramine (RDX)). It can be observed that the energy storage per unit mass of TiH_2_ is much higher than that of high explosives. Therefore, it has high military value and broad application prospects in the military field.
4. The chemical property is stable. It has good compatibility with strong oxidizing substances, long mixed storage time and almost no decomposition. Based on the above advantages, in recent years, some scholars have introduced titanium hydride (TiH_2_) as a high-energy additive into traditional energetic materials, such as explosives, propellants and pyrotechnics, and carried out a series of related research and made remarkable achievements in terms of explosive explosion performance, propellant burning rate and pyrotechnics explosion performance [26,27,28,29].

Recently, Yu [30,31] introduced TiH_2_ into Al/PTFE energetic materials for the first time under zero oxygen balance and mainly focused on the influence of different TiH_2_ content on the static mechanical properties of Al/PTFE/TiH_2_ composites under an equilibrium state. By using quasi-static compression experiments, the influence rules of different TiH_2_ content on the mechanical properties and reaction characteristics of Al/PTFE were compared and analyzed. It was found that, when adding TiH_2_ with a content of 5% to Al/PTFE material, the mechanical strength of Al/PTFE/TiH_2_ material is the largest and is 108 MPa, that of which is 15.1% higher than that of Al/PTFE material, and the probability of material reaction is 90%. The calorific value of the combustion of Al/PTFE/TiH_2_ material increases with the increase in the content of TiH_2_ under zero oxygen balance. The calorific value of combustion of Al/PTFE/TiH_2_ material with 0% the content of TiH_2_ is the smallest and is 13.81 MJ/kg. The calorific value of combustion of Al/PTFE/TiH_2_ material with 30% the content of TiH_2_ is the highest and is 16.15 MJ/kg. Cao [32] used TG/DSC to conduct thermal analysis of four kinds of composites, which are Al/PTFE, TiH_2_/PTFE, Al/TiH_2_ and Al/TiH_2_/PTFE only in the equilibrium state, and obtained the TG/DSC thermal analysis curve. Since there is no phase analysis for each peak, only a brief analysis for each peak of TG/DSC curve is made. Therefore, the drawn conclusion could not be used as a consistent conclusion. Although the content of TiH_2_ has a significant effect on the mechanical properties and reaction characteristics of Al/PTFE active materials, the jet formed by the Al/PTFE active cover under zero oxygen balance is easy to diverge, which is not conducive to the penetration and perforation of the target, while excessive Al can enhance the cohesiveness of the Al/PTFE active jet, thus improving the penetration effect of the target. Therefore, in order to achieve breakthroughs in the technology of Al-based polytetrafluoroethylene (PTFE) active materials under excess Al, it is necessary to clarify the reaction mechanism of Al based PTFE active materials under excess Al.

Based on the above, in order to fully understand the mechanism of Al-rich Al/PTFE/TiH_2_ active materials, Al/PTFE/TiH_2_, Al/TiH_2_, PTFE/TiH_2_ and Al/PTFE composite powders with different TiH_2_ contents were prepared in this study, and at the same time it is equipped with PTFE, Al and TiH_2_ single powder. The pyrolysis behavior of materials was investigated by thermogravimetric/differential scanning calorimetry (TG/DSC), and the phase analysis (XRD) of the reaction products at the corresponding peak temperature on the DSC curve of each material was carried out by X-ray diffraction (XRD). In addition, the calorific values of the reactions of PTFE/TiH_2_, Al/TiH_2_, Al/PTFE and Al/PTFE/TiH_2_ with different content of TiH_2_ were measured by an oxygen bomb calorimeter, and the phase analysis of residues of oxygen bomb experiment was carried out by X-ray diffraction (XRD). The chemical reaction mechanism of energetic materials is revealed, and the thermal properties, physical properties, mechanical properties and stability of various components and combination components of the active material are mastered. The chemical reaction mechanism of energetic materials is revealed, and the thermal properties, physical properties, mechanical properties and stability of various components and combination components of the active material are mastered. The results are expected to provide a practical reference for the safe nonreaction of active materials in the service operations of ammunition engineering, such as production and processing, transportation and storage, combat use and so on.

## 2. Materials and Methods

### 2.1. Materials

Detailed information on chemical substances used in this study is shown in Table 1.

The physical and chemical properties of raw materials are listed in Table 2.

### 2.2. Main Equipment

Thermogravimetric/Differential Scanning Calorimeter: STA449C, Netzsch, Germany. 

Scanning electron microscope: SEM450, FEI Nova Nano, USA.

X-ray diffractometer: Smartlab9, RIGAKU, Japan.

Digital oxygen bomb calorimetry analyzer: XRY-1A, Changji, China, oxygen atmosphere, calorific value measurement accuracy: less than 0.2%, oxygen bomb pressure test: 3 MPa, the reaction calorific value of the calorimeter is calibrated by burning benzoic acid in the calorimeter with the pressure set to 3 Mpa.

### 2.3. Sample Preparation

The composition ratios of materials for TG/DSC experiments are listed in Table 3, and the composition ratios of materials for oxygen bomb experiments are listed in Table 4.

The sample preparation has the following steps:

1. According to the mass ratio in Table 3 and Table 4, the powder is placed into the beaker after weighing with the electronic scale. At this time, an appropriate amount of anhydrous ethanol is added into the beaker while continuously stirring for about 30 min, and an approximate fully mixed solution is created. The beaker containing the mixed solution was dried in a vacuum drying oven at 55 °C for 48 h in order to obtain a solid mixture of fully mixed bulk materials.
2. The solid mixture of the block material is pounded with a glass rod and continuously stirred into a powder.

**Figure 2:**
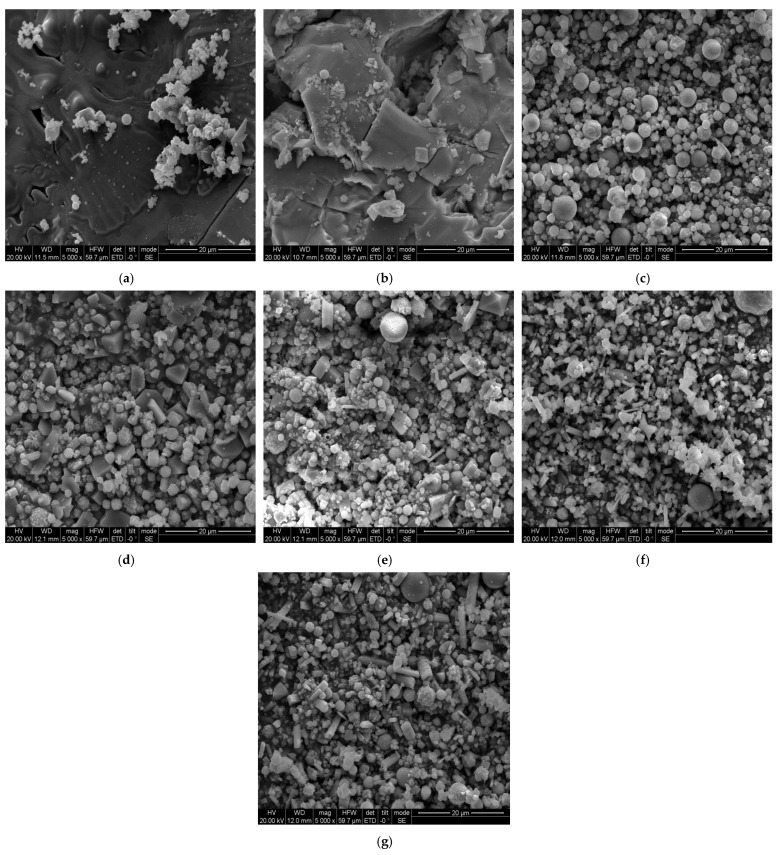
SEM of initial microstructure of raw materials. (**a**) SEM diagram of pure PTFE powder; (**b**) SEM diagram of pure TiH_2_ powder; (**c**) SEM diagram of pure Al powder; (**d**) SEM diagram of Al/TiH_2_ powder; (**e**) SEM diagram of PTFE/TiH_2_ powder; (**f**) SEM diagram of Al/PTFE powder; (**g**) SEM diagram of Al/TFE/TiH_2_ powder.

Before the experiment, in order to check the mixing homogeneity of Al/TiH_2_, PTFE/TiH_2_, Al/PTFE and Al/PTFE/TiH_2_ samples components, the scanning electron microscope (SEM) of FEINova 450 was used to observe the microstructure of them. The results are shown in Figure 2.

It can be observed from Figure 2 that PTFE particles with an average particle size of 27 μm present soft and irregular shapes. TiH_2_ particles with an average diameter of 4–6 μm show firm and hard irregular surface polygon shape. Al particles with an average diameter of 6–7 μm are regular spherical, and their hardness is between that of PTFE particles and that of TiH_2_ particles. As shown in Figure 2d–f, the results show that each component of the material is mixed more evenly. As shown in Figure 2g, the results show that Al particles and TiH_2_ particles are uniformly distributed in PTFE base, indicating that all the components of the material are uniformly mixed during the preparation process.

### 2.4. Experimental Content

The pyrolysis behavior and reaction process of samples 1–7 were analyzed by TG-DSC. The samples with an average mass of 2 mg are placed in the Al_2_O_3_ crucible of TG/DSC. In order to prevent air from participating in the reaction, experiments were carried out in high purity argon (99.999%) with a flow rate of 40 mL/min. The program of the device was set at a heating rate of 5 °C/min, and the temperature range was at room temperature to 1000 °C. 

The reaction calorific values of samples 1#–7# were measured by an oxygen bomb calorimeter in the atmosphere of high purity oxygen (99.999%). The calorific value of the calorimeter was calibrated by burning benzoic acid in a calorimeter with a pressure setting of 3 MPa.

The X-ray diffraction (XRD) test system was used to detect the composition of the residue of samples 1–7 after thermal analysis experiment, the residue at each peak temperature of TG/DSC curve of samples 1–7 and the residue of samples 1#–7# after the oxygen bomb experiment. The instrument parameters were set as follows: the tube voltage was 40 kV; the current was 150 mA; Cu-k_α_ radiation (λ = 0.15416nm); the scanning range 2θ was 10–90°; the scanning step was 0.02°; and the scanning speed was 4 °/min.

## 3. Results and Discussion

For the TG/DSC curves of the following various materials, the peak shape upward indicates endothermic reaction, and the peak shape downward indicates exothermic reaction.

### 3.1. Pyrolysis Behavior and Reaction Process of Polytetrafluoroethylene (PTFE)

**Figure 3:**
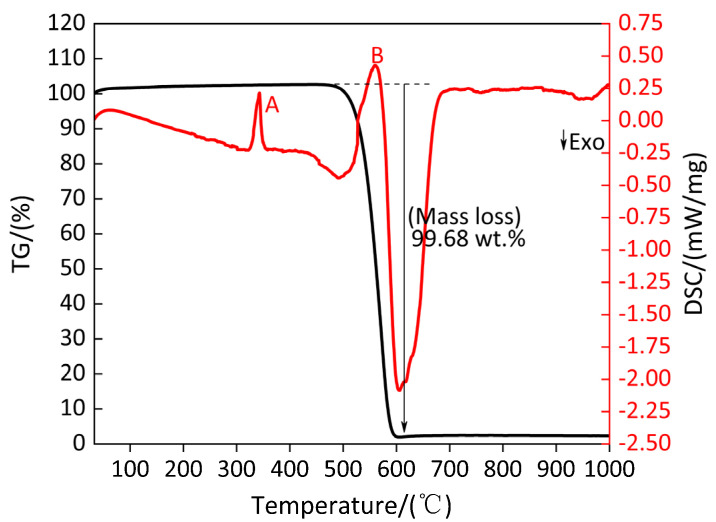
TG/DSC curve of PTFE. A and B: Endothermic peak.

Figure 3 shows the TG/DSC curve of PTFE. The parameters of peak A and peak B on DSC curve of PTFE are listed in Table 5. It can be observed from the DSC curve in Figure 3 that the endothermic phenomenon of PTFE occurs twice during heating to 1000 °C, corresponding to the endothermic peak A and endothermic peak B on the DSC curve. As shown in Table 5, the endothermic peak A begins at 330.9 °C, terminates at 347.7 °C and the peak temperature is 342.2 °C, which is close to the melting point of PTFE used in this experiment and the corresponding absorption heat of this peak is 63.07 J/g. Within the temperature range of this peak, the TG curve remained stable, indicating that the quality of PTFE did not change; thus, peak A was the endothermic melting peak of PTFE. In the endothermic peak B of DSC curve, when the temperature increases from 527.0 °C to the peak temperature of 560.1 °C, the corresponding TG curve begins to decrease from a slow decline to a sharp decline. When the temperature increases from the peak temperature to the end temperature of 596.2 °C, the corresponding TG curve changes from a sharp decline to a slow decline. The weight loss of PTFE is as high as 99.68%, and the absorbed heat is 1265 J/g, indicating that endothermic peak B is the decomposition endothermic peak of PTFE. After that, as the temperature continues to increase, the weightlessness curve was basically parallel to the temperature axis, indicating that the pyrolysis of PTFE is basically completed when the temperature reached 596.2 °C.

Figure 3 shows that the DSC curve is smooth, all the corresponding points are negative in the low temperature range of 100–300 °C and the temperature range was at 400–500 °C, indicating that PTFE absorbs heat evenly and steadily. In the temperature range of 300–400 °C, the first thermal peak A appears in PTFE, which corresponds to the phase change of PTFE, which is consistent with the statement in Reference [33] that “when PTFE is heated to 327–340 °C, the crystalline region of PTFE is destroyed and begins to transform into transparent colloid with amorphous structure”. There is a large thermal peak B in the high temperature range of 500–600 °C, which corresponds to the temperature range of the TG curve of weightlessness, indicating that the pyrolysis of PTFE is an apparent endothermic reaction. Based on the above analysis, the pyrolysis process of PTFE can be summarized as follows: in argon atmosphere, the pyrolysis of PTFE is an endothermic reaction, and the whole pyrolysis process only has one weightlessness stage, which indicates that the pyrolysis process of PTFE is a one-step reaction.

The average bond energy of C–C bond in PTFE molecular structure is less than that of the C–F bond, and the energy of terminal the –CF_2_–CF_2_· bond produced by the long straight chain structure of PTFE and random fracture of similar bond is less than that of intermediate –CF_2_–CF_2_· bond; thus, the C–C bond fractures easier than the C–F bond. Therefore, two fluorocarbenes (CF_2_:) can be easily formed from this fragment. The carbene group easily forms the gaseous product tetrafluoroethylene (TFE, CF_2_ = CF_2_), which is stated as follows.

$$CF_{2}:\overset{CF_{2}:}{\to }CF_{2}=CF_{2}$$

When TFE accumulates to a certain amount, the carbene group will react with it to form a small molecule gas compound hexafluoropropylene (HFP, CF_3_-CF = CF_2_), which is stated as follows.

$$CF_{2}=CF_{2}\overset{CF_{2}:}{\to }CF_{3}–CF=CF_{2}$$

After the formation of HFP, it can react with carbene group to form gas octafluorocyclobutene (OFIB, $CF_{3}–\underset{CF_{3}}{\underset{|}{C}}=CF_{2}$) and gas octafluorobutene (OFB, CF_3_–CF=CF–CF_3_), which is described as follows.

$$2CF_{3}–CF=CF_{2}\overset{2CF_{2}:}{\to }CF_{3}–\underset{\underset{CF_{3}}{|}}{C}=CF_{2}+CF_{3}–CF=CF–CF_{3}$$

In the temperature range of 500–600 °C, as OFIB and OFB continue to decompose and fracture, they eventually produce carbon black (C) and gas products (carbon tetrafluoride (CF_4_), hexafluoroethane (C_2_F_6_)) and low molecular weight PTFE ((CF_2_)_n_). The TFE can also form octafluorocyclobutane (OFCB, $\begin{array}{ccc} CF_{2} & – & CF_{2}\\ | & & |\\ CF_{2} & – & CF_{2} \end{array}$) by dimerization, which is described as follows.

$$2CF_{2}=CF_{2}\overset{\mathrm{Dimerization}}{\to }\begin{array}{ccc} CF_{2} & – & CF_{2}\\ | & & |\\ CF_{2} & – & CF_{2} \end{array}$$

**Figure 4:**
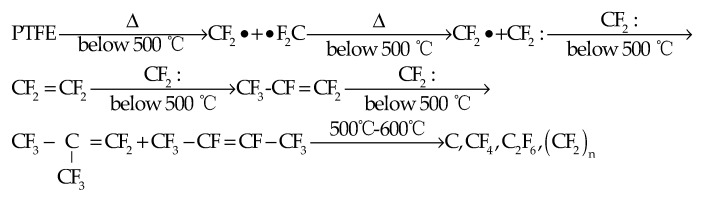
Structural framework diagram of pyrolysis mechanism of PTFE.

Based on the above analysis, the pyrolysis mechanism of PTFE is shown in Figure 4.

**Figure 5:**
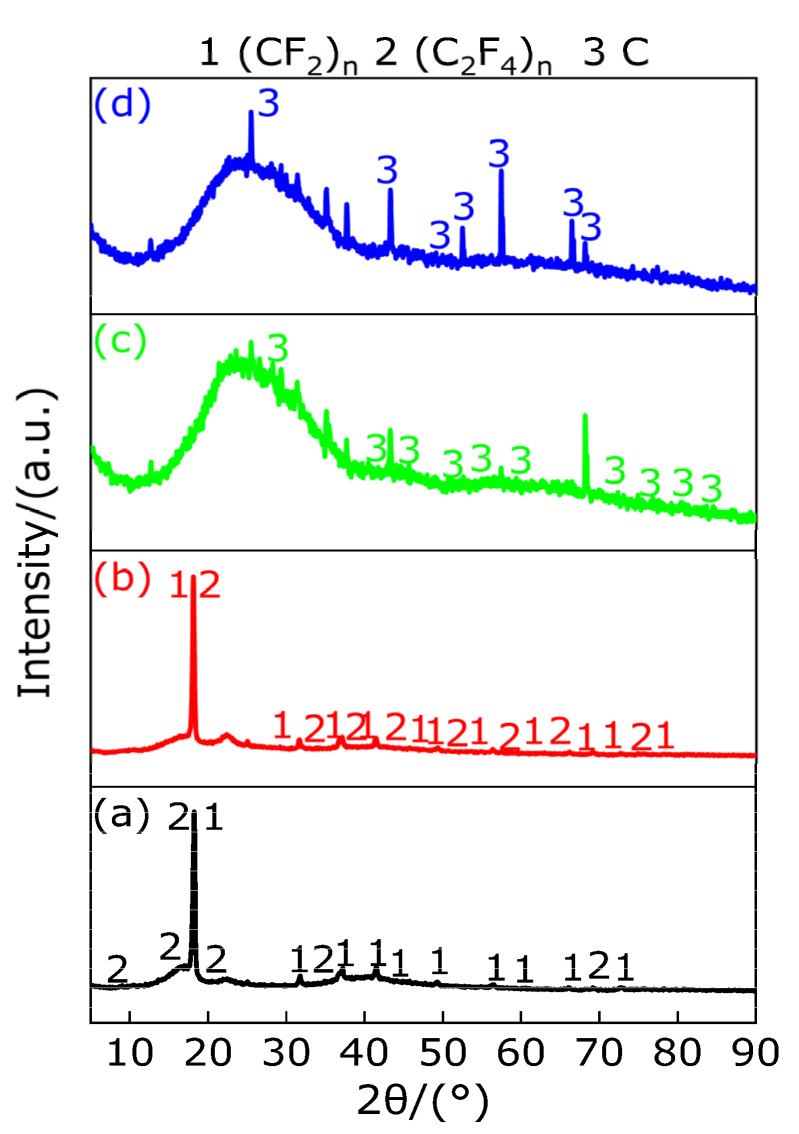
XRD pattern of PTFE at main temperature. (**a**) XRD pattern at 342.2 °C; (**b**) XRD pattern at 560.1 °C; (**c**) XRD pattern at 605.9 °C; (**d**) XRD pattern at 1000 °C.

The reaction mechanism of material is determined by the composition of the residue at the endothermic and exothermic peaks. Therefore, in order to fully understand the pyrolysis reaction mechanism of PTFE, it is necessary to detect the composition of the residues at each peak temperature of PTFE. In order to verify whether the reaction of PTFE at 596.2 °C is basically completed and to test the composition of PTFE pyrolysis products after 600 °C, the composition of the material at 605.9 °C and 1000 °C is determined by X-ray diffraction (XRD), and the XRD patterns of the material at the two peak temperatures were given, as shown in Figure 5. It can be observed from Figure 5a that the composition of PTFE at the peak temperature of 342.2 °C is of low molecular weight PTFE ((CF_2_)_n_), large molecular weight PTFE ((C_2_F_4_)_n_) and a long chain of PTFE, which indicates that only the crystalline region of PTFE gradually transforms into an amorphous region at this temperature, and a small amount of long chain fracture of PTFE produces undecomposed (CF_2_)_n_ and (C_2_F_4_)_n_ with different molecular weight. Therefore, there is no pyrolysis reaction occurring in the temperature range of 330.9–347.7 °C, but part of the PTFE long chain breaks. It can be observed from Figure 5b that PTFE mainly contains low molecular weight PTFE ((CF_2_)_n_) and a small part of large molecular weight PTFE ((C_2_F_4_)_n_) at the peak temperature of 560.1 °C, which indicates that the unbroken long chain of PTFE is completely broken at the temperature range of 527.0–560.1 °C in order to obtain large and small molecular weights of (C_2_F_4_)_n_ and (CF_2_)_n_. When the temperature increased from 560.1 °C to 605.9 °C, these PTFE with large and small molecular weight can be pyrolyzed quickly in order to produce gas and solid carbon black. It is also known from Figure 5c,d that the composition of the residue is only carbon black in the temperature range of 605.9–1000 °C, which indicates that PTFE is completely pyrolyzed and produces a large amount of gas in the temperature range of 500–600 °C. When the temperature is higher than 600 °C, the diffraction peak of XRD pattern is almost unchanged, indicating that when the temperature is higher than 600 °C, and that PTFE pyrolysis produces carbon black with the same material. Based on the above analysis, the thermal decomposition products of PTFE in different temperature ranges are listed in Table 6.

### 3.2. Pyrolysis Behavior and Reaction Process of Titanium Hydride (TiH_2_)

**Figure 6:**
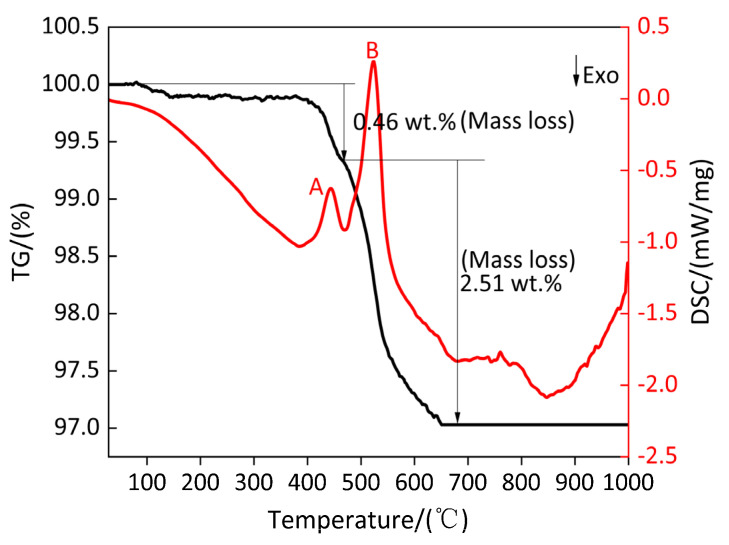
TG/DSC curve of TiH_2_ pyrolysis process. A and B: Endothermic peak.

Figure 6 shows the TG/DSC curve of the pyrolysis process of TiH_2_. Table 7 shows the parameters of peak A and peak B on the DSC curve of TiH_2_. Figure 6 and Table 7 show that the endothermic peak A and endothermic peak B appear on the DSC curve when TiH_2_ is decomposed by heating. For peak A and peak B, the corresponding starting temperatures are 386.33 °C and 470.33 °C, the corresponding peak temperatures are 442.9 °C and 523.8 °C, the corresponding termination temperatures are 470.33 °C and 650.33 °C and the corresponding absorption heat values are 109.2 J/g and 651.7 J/g. When the temperature is in the range of 100–463.7 °C, the mass curve decreases slightly, but the decrease range is very small and is 0.46%, which is caused by the first dehydrogenation of TiH_2_ during the endothermic process. When the temperature is in the range of 470.33–523.8 °C, the mass curve decreases sharply. When the temperature is in the range of 523.8–650.33 °C, the mass curve decreases slowly. This is due to the second dehydrogenation initiated by TiH_2_ through high temperature endothermic on the basis of the first dehydrogenation. The mass loss caused by the second dehydrogenation was 2.51%. The mass loss caused by the second dehydrogenation accounted for 84.51% of the total mass loss. The total loss caused by dehydrogenation is 2.97%, which is less than the theoretical dehydrogenation amount of 4.01%, which may be related to the purity of raw material, instrument error or oxidation. Based on the above analysis, the pyrolysis reaction of TiH_2_ in Ar atmosphere is a multi-stage reaction rather than a one-step reaction.

**Figure 7:**
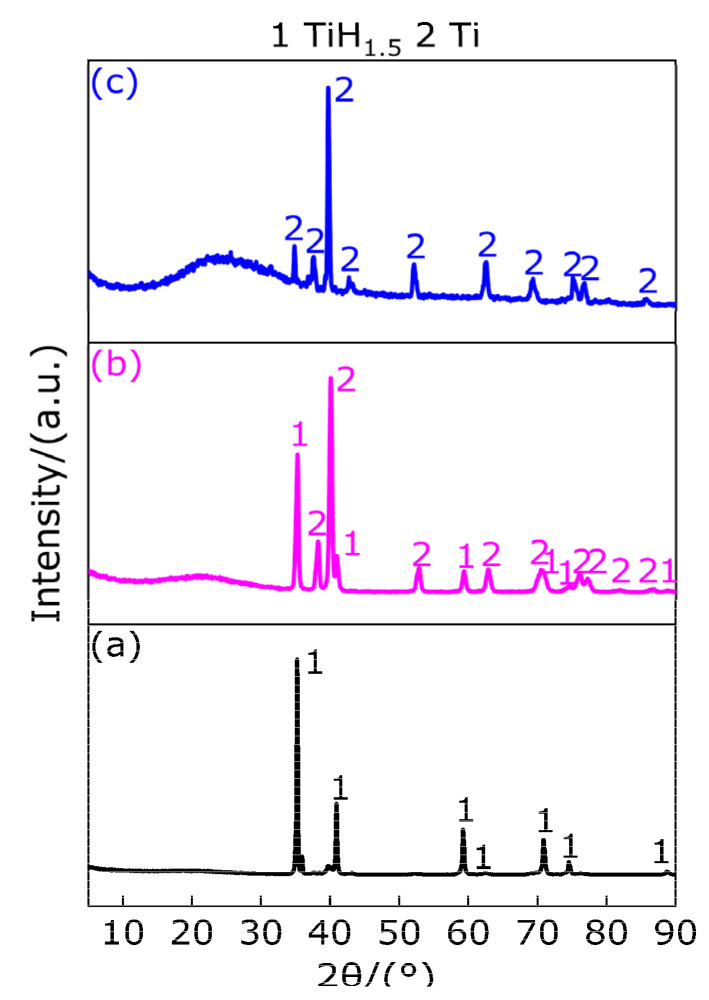
XRD patterns of TiH_2_ at peak temperature and 1000 °C. (**a**) XRD pattern at 442.9 °C; (**b**) XRD pattern at 523.8 °C; (**c**) XRD pattern at 1000 °C.

Figure 7 shows the XRD patterns at the peak temperature and at 1000 °C. It can be observed from Figure 7a–c that there is a TiH_1.5_ diffraction peak at the peak temperature of 442.9 °C, which indicates that TiH_2_ is not dehydrogenated when the temperature is less than 386.33 °C, and the corresponding TG curve shows that the quality of the sample decreases slightly, which may be caused by the removal of impurities in the TiH_2_ sample. When the temperature is in the range of 386.33–442.9 °C, half of the hydrogen is removed from TiH_2_ for the first time, and TiH_1.5_ exists in the temperature range of 386.33–470.33 °C. The XRD patterns show that TiH_1.5_ and Ti diffraction peaks exist at the peak temperature of 523.8 °C, indicating that 1.5 hydrogen is removed from partial TiH_1.5_ to produce Ti in the temperature range of 470.33–523.8 °C. In the temperature range of 523.8–650.33 °C, the undecomposed TiH_1.5_ loses 1.5 hydrogen and then produces Ti due to high temperature endothermic. When the temperature is at 1000 °C, only Ti diffraction peaks are in the XRD pattern, indicating that only Ti matter exists at the temperature range of 650.33–1000 °C. To sum up the above, in the argon atmosphere the substances produced by TiH_2_ at different temperature ranges are listed in Table 8. According to the above analysis, the conclusion is consistent with Reference [35] in that the phase transformation process of TiH_2_ heating dehydrogenation in argon atmosphere is TiH_2_ → TiH_1.5_ → Ti, which indicates that the thermal decomposition of TiH_2_ in argon atmosphere does not directly produce Ti and hydrogen. The Bhosle et al. [36] study shows that TiH_2_ produces a stable phase TiH_x_ (0 < x ≤ 2) at high temperatures and releases x hydrogen (H), and hydrogen (H) combines with hydrogen (H) to form hydrogen (H_2_). The specific equations follow Equations (1), (2) and (3). Hydrogen (H) is a very active element. With the increase in temperature, H diffuses rapidly in the form of H^+^ in metal. Since the atomic ratio of hydrogen/titanium (H/Ti) is very high and the content of free H^+^ is also very high, it is easy to overcome the chemical bond of TiH_x_ (0 < x ≤ 2) and to escape from the metal Ti [37].

TiH_2_ → TiH_x_ + (2 − x)H (1)

TiH_x_ → Ti + xH (2)

H + H → H_2_ (3)

The diffusion state of hydrogen (H) in metal Ti can be given by the diffusion coefficient D [38]:

(4) $$D=1.8\times 10^{-2}\exp\left(-\frac{12380}{RT}\right)$$

where D is the diffusion coefficient of hydrogen (H) in metal Ti, cm^2^/s; R is the gas constant, 8.314 J/(mol·K); and T is the heating temperature, °C.

**Figure 8:**
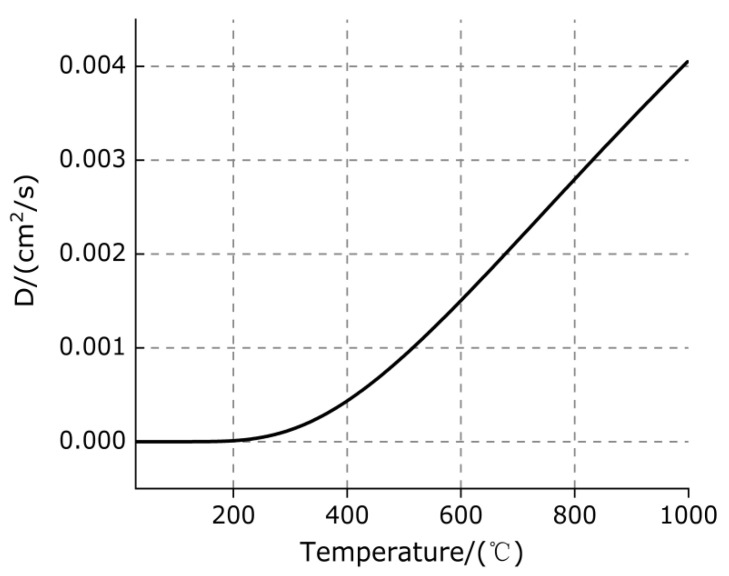
The diffusion of H in metal Ti varies with temperature.

The diffusion of H in metal Ti varies with temperature can be obtained by Equation (4), as shown in Figure 8.

It can be observed from Figure 8 that when the temperature is less than 400 °C, the diffusion of hydrogen (H) in Ti is extremely slow and is not significantly affected by the temperature; when the temperature exceeds 400 °C, the diffusion of hydrogen in titanium is significantly affected by temperature. The diffusion of hydrogen (H) in titanium (Ti) constantly increases with the increase in heating temperature. 

Based on the above analysis results and XRD test results, the change rule of the continuous decomposition of TiH_2_ is basically reflected; that is, the heating decomposition of TiH_2_ in argon atmosphere can be divided into three stages. In the first stage, TiH_2_ does not decompose, but the mass loss may be caused by the removal of impurities in TiH_2_ sample. In the second stage, TiH_2_ lost half of its hydrogen and converted to TiH_1.5_. In the third stage, TiH_1.5_ lost 1.5 hydrogen and converted to Ti. The corresponding three stages of temperature, decomposition time, loss quality and other parameters are shown in Table 9. The sample mass corresponding to the initial temperature of each stage is the initial mass m_1_, the sample mass corresponding to the termination temperature is the termination mass m_2_, and the loss mass Δm is the initial mass m_1_ minus the termination mass m_2_. When the temperature is T, the mass of the sample is m(T). The percentage of mass loss (α(T)) can be given by the following formula.

(5) $$\alpha \left(T\right)=\left|\frac{m\left(T\right)-m_{1}}{m_{1}-m_{2}}\right|$$

**Figure 9:**
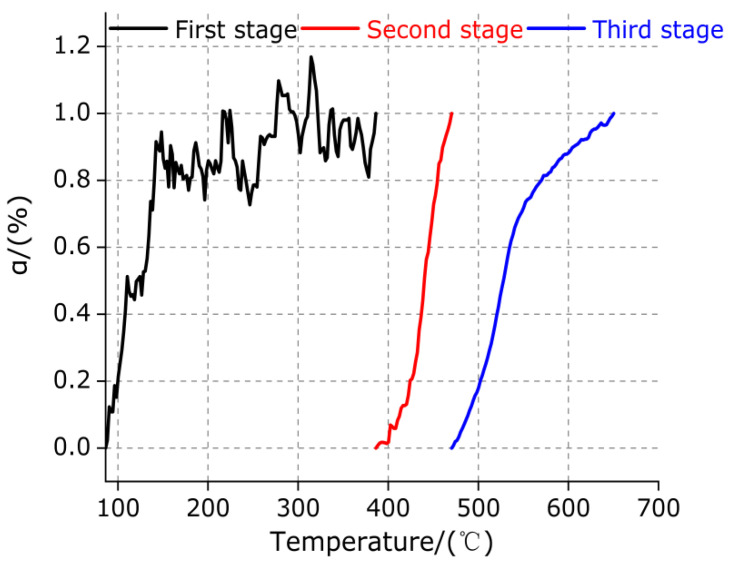
Variation trend of percentage of mass loss (α) with temperature in each stage of TiH_2_.

**Figure 10:**
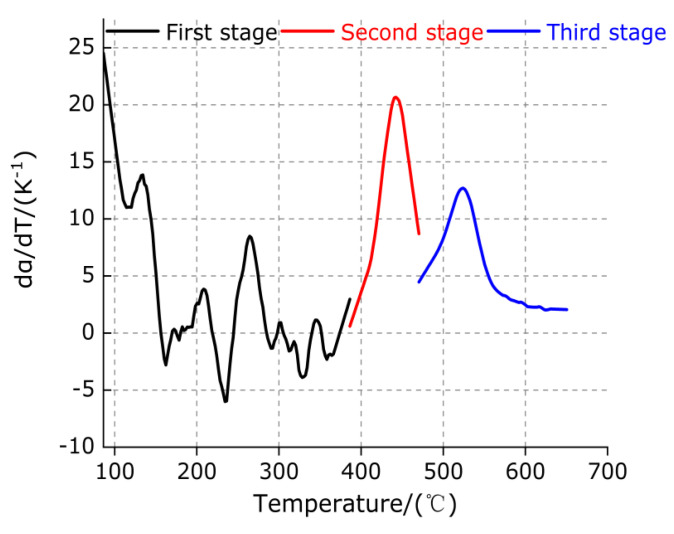
DTG curve of TiH_2_ in each stage.

The change trend of the percentage of mass loss (α(T)) with temperature at each stage is calculated by Formula (5), as shown in Figure 9. The differential treatment of the first derivative of the curves in Figure 9 can be obtained by the DTG (dα/dT) curve of each stage, as shown in Figure 10.

It can be observed from Figure 9 and Figure 10 that, in the first stage (86.33–386.33 °C), the percentage of mass loss (α(T)) gradually increases slowly with the increase in temperature T, indicating that the mass loss rate (dα/dT) of TiH_2_ gradually decreases, which is consistent with the slow diffusion of H in metal Ti at low temperature. The percentage of mass loss (α(T)) of the second stage (386.33–470.33 °C) and the third stage (470.33–650.33 °C) increases slowly at first and then increases rapidly with the increase in temperature T, which indicates that the mass loss rate (dα/dT) of TiH_2_ in these two stages first increases and then decreases with the increase in temperature, and the maximum mass loss rate of the second stage is higher than that of the third stage. It is further explained that the content of endothermic dissociated hydrogen in TiH_2_ is higher than that of the third stage, the decomposition of TiH_2_ gradually increases starting from the second stage, and the decomposition of TiH_2_ is completed when the temperature reaches a certain high temperature.

The decomposition enthalpies of Ti and H and the heat capacities of Ti, H_2_ and TiH_2_ in the thermal decomposition reaction formula TiH_2_ → Ti + H_2_ can be obtained from Reference [39]. The enthalpies of the decomposition reactions of titanium (Ti) and hydrogen (H) are listed in Table 10. The heat capacities of Ti, H_2_ and TiH_2_ are listed in Table 11.

The equilibrium constant of thermal decomposition reaction at a certain temperature can be obtained by following the Kirchhoff formula and van’t Hoff equation integral [40].

(6) $$\Delta_{r}H_{m}^{0}\left(T\right)=\Delta_{r}H_{m}^{0}\left(298.15K\right)+\int_{298.15}^{T}\left[C_{m}\left(TiH_{2}\right)-C_{m}\left(Ti\right)-C_{m}\left(H_{2}\right)\right]dT$$

(7) $$\int_{K\left(298.15K\right)}^{K\left(T\right)}d\ln K=\int_{298.15}^{T}\left[\Delta_{r}H_{m}^{0}\left(T\right)/RT^{2}\right]dT$$

Combined with Table 10, when the temperature is 298.15 K, the equilibrium constant K (298.15 K) of the thermal decomposition reaction is as follows.

(8) $$K\left(298.15K\right)=\exp\left[-\Delta_{r}G_{m}^{0}\left(298.15K\right)/RT\right]=1.186\times 10^{14}$$

Combined with Table 10 and Table 11 and Formulas (6)–(8), the relationship between the thermal decomposition equilibrium constant K(T) and temperature T can be obtained.

(9) $$K\left(T\right)=\exp\left(1.837+1.359\times 10^{4}T^{-1}-2.619\ln T-2.614\times 10^{-4}T+6.545\times 10^{-9}T^{2}-6.671\times 10^{-11}T^{3}\right)$$

According to the equation of state of ideal gas, the relationship between the decomposition equilibrium hydrogen pressure ($P_{H_{2}}$ and MPa) of gaseous H_2_ and the equilibrium constant K(T) of thermal decomposition reaction can be obtained; the relationship is as follows.

(10) $$P_{H_{2}}=\frac{1}{K\left(T\right)}$$

The relationship between equilibrium hydrogen pressure $P_{H_{2}}$(MPa) and temperature T (°C) can be obtained from Equations (9) and (10).

(11) $$P_{H_{2}}=\exp\left(-1.837-1.359\times 10^{4}T^{-1}+2.619\ln T+2.614\times 10^{-4}T-6.545\times 10^{-9}T^{2}+6.671\times 10^{-11}T^{3}\right)$$

**Figure 11:**
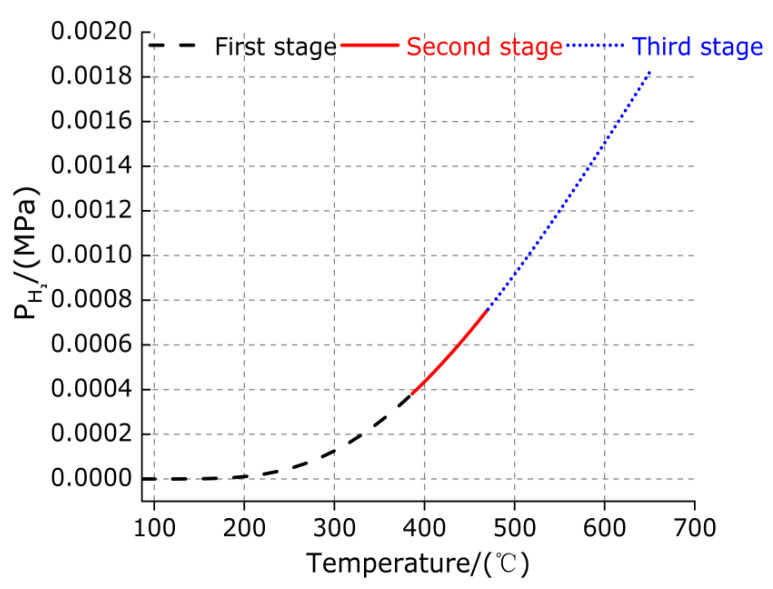
The variation curve of equilibrium hydrogen pressure of decomposition for TiH_2_ in three stages with temperature.

The variation trend of equilibrium hydrogen pressure of decomposition for TiH_2_ in three stages with temperature can be obtained from Equation (11), as shown in Figure 11.

It can be observed from Figure 11 that for the first stage (86.33–386.33 °C), when the temperature is less than 200 °C, the equilibrium hydrogen pressure of thermal decomposition is 0 Pa. When the temperature increased from 200 °C to 386.33 °C, the equilibrium hydrogen pressure of thermal decomposition increased slowly, but the increment is very small, which may be caused by the elimination of impurities in TiH_2_ samples. For the second stage (386.33–470.33 °C), with the increase in temperature, the equilibrium hydrogen pressure of thermal decomposition gradually increases, but the increment is not large, which be caused by the loss of half hydrogen of TiH_2_ endothermic for the first time. For the third stage (470.33–650.33 °C), with the increase in temperature, the equilibrium hydrogen pressure of thermal decomposition increases gradually and the increment is very large, which is caused by partial TiH_1.5_ decomposition. The equilibrium hydrogen pressure of thermal decomposition is only about 126.48 Pa at 300 °C, but it increases significantly to 1.82 kPa at 650.33 °C, and it has a tendency to accelerate rise with the increase of temperature, which is caused by the decomposition of TiH_1.5_ without the complete loss of hydrogen with the increase of temperature. To sum up, from the first stage to the third stage, the equilibrium hydrogen pressure of thermal decomposition increases gradually, which indicates that the degree of the thermal decomposition reaction of TiH_2_ decreases gradually to the end with the temperature increasing to 650.33 °C. Based on the above, it can be observed that the decomposition of TiH_2_ can be used as an effective method to produce high purity hydrogen with high efficiency.

### 3.3. Pyrolysis Behavior of Aluminum (Al)

**Figure 12:**
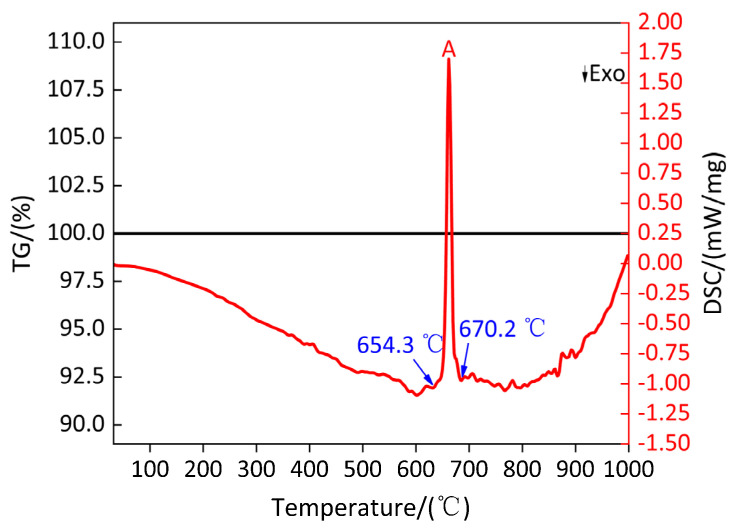
TG/DSC curve of sample Al. A: Endothermic peak.

Figure 12 shows the TG/DSC curve of Al. Table 12 shows the parameters of peak A on the DSC curve for Al. It can be observed from Figure 12 that in argon atmospheres, when the temperature is heated to 1000 °C, the material Al appears as an endothermic phenomenon, which corresponds to the endothermic peak A on the DSC curve. It can be observed from Table 12 that the temperature range of endothermic peak A is 654.3–670.2 °C. In this temperature range, the mass of sample Al does not change, indicating that peak A is the melting endothermic peak of Al. In the entire heating temperature range, the mass of sample Al remains unchanged, the peak temperature of the sample Al was 661.7 °C, which was close to the melting point of Al at 660 °C, and the total absorption heat of sample Al is 299.6 J/g. When the temperature is lower than 661.7 °C, the sample Al is still solid during the endothermic process with the increase in temperature. When the temperature is not less than 661.7 °C and not more than 1000 °C, with the increase in temperature and during the endothermic process, the sample Al begins to transform from a solid state to a solid–liquid mixed state and finally to a liquid state.

**Figure 13:**
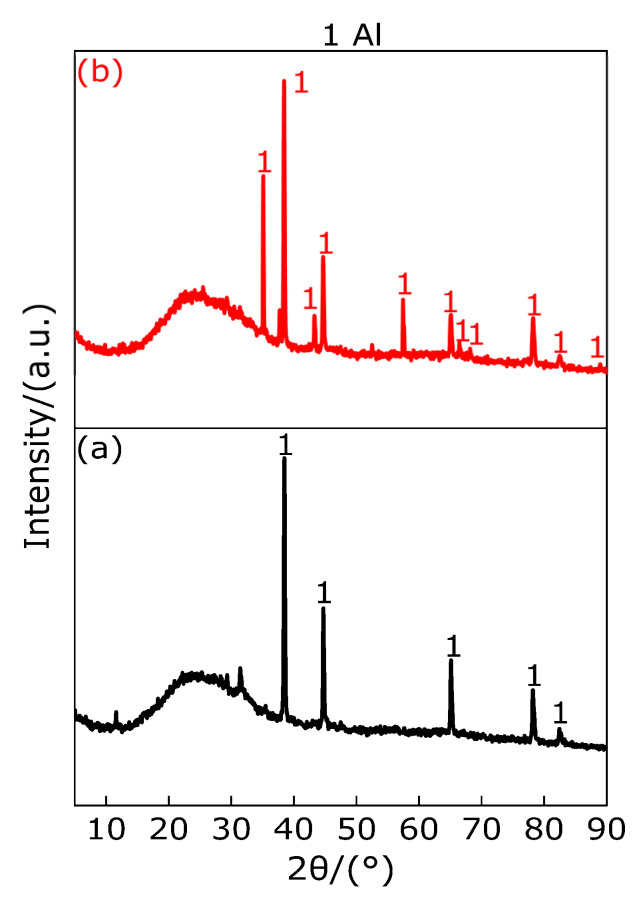
XRD patterns of sample Al at peak temperature and 1000 °C. (**a**) XRD pattern at 661.7 °C; (**b**) XRD pattern at 1000 °C.

In order to understand the pyrolysis mechanism of Al during heating process, the composition of the residues at peak temperatures of 661.7 °C and 1000 °C were analyzed by X-ray diffraction (XRD). The results are shown in Figure 13. It can be observed from Figure 13a,b that the substance Al exists at the peak temperature and 1000 °C, and no other substance exists, which indicates that when the temperature is heated from room temperature to 1000 °C, Al only experiences changes in the physical state without chemical changes in matter; that is, solid state → solid-liquid mixed state → liquid state.

### 3.4. Pyrolysis Behavior and Reaction Process of Aluminum/Titanium Hydride (Al/TiH_2_)

**Figure 14:**
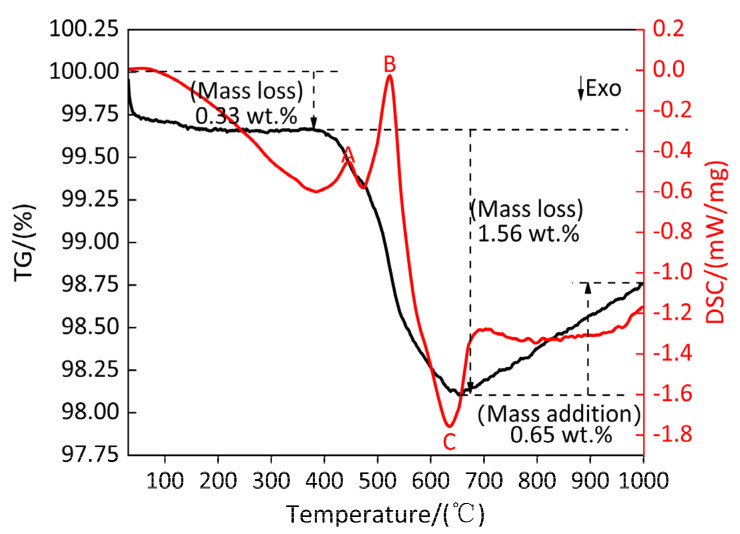
TG/DSC curves of Al/TiH_2_ pyrolysis. A and B: Endothermic peak; C: Exothermic peak.

Figure 14 shows the TG/DSC curves of Al/TiH_2_ pyrolysis. Table 13 demonstrate the parameters of peak A, peak B and peak C on the DSC curve of sample (Al/TiH_2_). It can be observed from Figure 14 and Table 13 that a small endothermic peak, a larger endothermic peak and an exothermic peak appear on the curve of Al/TiH_2_, which are peak A, peak B and peak C, respectively. The temperature range of small peak A is 418.9–465.1 °C, and the corresponding absorption heat is 55.64 J/g. During the absorption heat period, the mass curve of sample (Al/TiH_2_) decreases, and the corresponding mass fraction increment of the sample is −0.33%, indicating that the small peak A is the decomposition endothermic peak of sample (Al/TiH_2_). The larger peak B starts at 491.3 °C and ends at 545.8 °C, and the corresponding absorption heat is 461.3 J/g. The heat causes the mass curve of the sample (Al/TiH_2_) to first decrease rapidly and then decrease slowly, and the corresponding mass fraction increment of sample is −1.56%, which indicates that the larger peak B is the decomposition endothermic peak of the sample (Al/TiH_2_). With the increase in temperature, an exothermic peak C appears on the DSC curve at the peak temperature of 633.8 °C, and the heat release begins at 598.4 °C, terminates at 671.2 °C, and releases heat measuring 389.7 J/g. During the exothermic period, the mass curve of the sample (Al/TiH_2_) rises and the corresponding mass fraction increment of the sample is 0.65%. The reason for this may be that Ti metal produced by dehydrogenation of TiH_2_ is oxidized. As the relative atomic mass difference between hydrogen (H) and oxygen (O) is very large, as long as the sample is slightly oxidized, the weight gain trend of the sample is very obvious [41].

**Figure 15:**
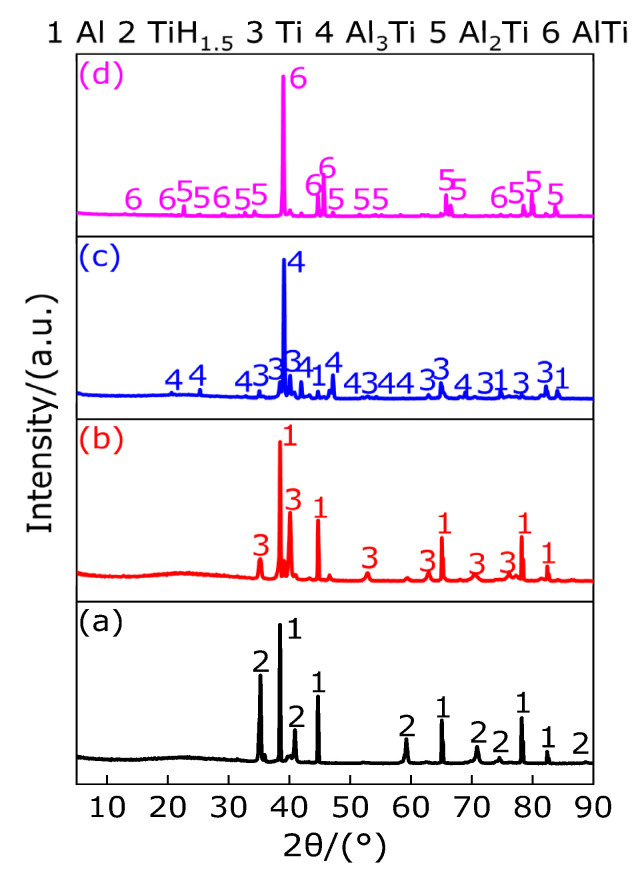
XRD patterns of the sample (Al/TiH_2_) at peak temperature and 1000 °C. (**a**) XRD pattern at 444.7 °C; (**b**) XRD pattern at 523.9 °C; (**c**) XRD pattern at 633.8 °C; (**d**) XRD pattern at 1000 °C.

Figure 15 shows the XRD patterns of the sample (Al/TiH_2_) at the peak temperature and 1000 °C in argon atmosphere. It can be observed from Figure 15 that, for peak A, there exists diffraction peaks of Al and TiH_1.5_ at the peak temperature of 444.7 °C. According to Section 3.2, TiH_2_ begins to dehydrogenate at 386.33 °C. Therefore, in the temperature range of 386.33–418.9 °C and 418.9–465.1 °C, the first dehydrogenation of TiH_2_ ends and transforms into TiH_1.5_. It is indicated that Al does not react with TiH_2_ in the temperature range of 386.33–465.1 °C, but TiH_2_ is dehydrogenated to produce TiH_1.5_, which coexists with Al. There exist diffraction peaks of Al and Ti at the peak temperature of 523.9 °C of endothermic peak B, which indicates that TiH_1.5_ decays 1.5 hydrogen and transforms into Ti, which coexists with Al in the temperature range of 491.3–545.8 °C. It is indicated that Al does not react with TiH_1.5_ in this temperature range, and only TiH_1.5_ dehydrogenation reaction occurred. When the temperature are in the range of 545.8−598.4 °C, Al and Ti still coexist. When the temperature is higher than 598.4 °C and lower than 671.2 °C, Al_3_Ti appears in the XRD pattern. Diffraction peaks of Al_2_Ti and AlTi exist in the temperature range of 671.2–1000 °C. Based on the above analysis, when the temperature is less than 598.4 °C, Al does not react with TiH_2_, and only TiH_2_ is dehydrogenated to Ti at two times. When the temperature increases from 598.4 °C to 671.2 °C, there still exists a new Al_3_Ti diffraction peak in addition to the diffraction peaks of Al and Ti, which indicates that parts of the Al and parts of the Ti reactions occur to produce Al_3_Ti in the temperature range of 598.4–671.2 °C. When the temperature increases from 671.2 °C to 1000 °C, there exist diffraction peaks of Al_2_Ti and AlTi, but no diffraction peak of Al_3_Ti is detected, which indicates that the disproportionation reaction of Al_3_Ti occurs to produce Al_2_Ti and Al or AlTi and Al, and finally Al reacts with Ti to produce Al_2_Ti or AlTi in the temperature range of 671.2–1000 °C. Based on the above analysis, the main chemical reactions occurring in the Al/TiH_2_ composite system under argon atmosphere are as follows (12)–(17).

TiH_2_ → TiH_1.5_ + 1/2H (12)

TiH_1.5_ → Ti + 1.5H (13)

H + H → H_2_(g) (14)

3Al + Ti → Al_3_Ti (15)

Al_3_Ti → Al_2_Ti + Al or Al_3_Ti → AlTi + 2Al (16)

Al + Ti → AlTi or 2Al + Ti → Al_2_Ti (17)

According to the conclusion obtained in Section 3.2 and the above analysis, the substances produced in sample (Al/TiH_2_) at different temperatures under argon atmosphere can be listed in Table 14.

### 3.5. Pyrolysis Behavior and Reaction Process of Polytetrafluoroethylene/Titanium Hydride (PTFE/TiH_2_)

**Figure 16:**
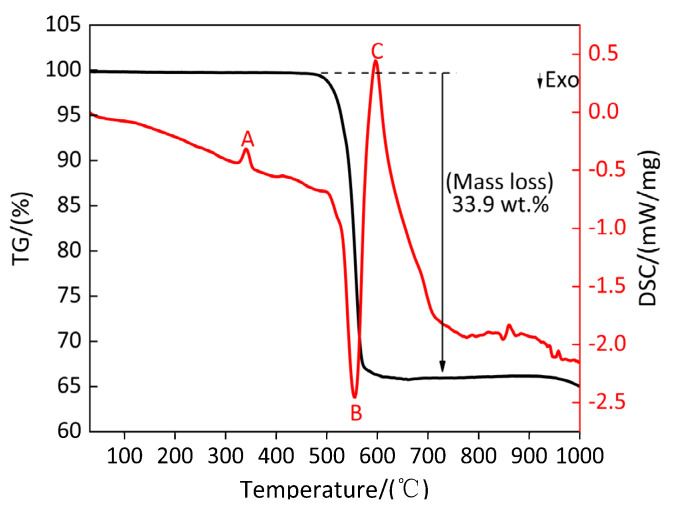
TG/DSC curve of PTFE/TiH_2_ composite. A and C: Endothermic; B: Exothermic peak.

Figure 16 shows the TG/DSC curve of the sample (PTFE/TiH_2_). Table 15 shows the parameters of peak A, B and C on DSC curve of sample (PTFE/TiH_2_). It can be observed from Figure 16 and Table 15 that the small peak A is the melting endothermic peak of PTFE. The endothermic peak starts at 328.5 °C and ends at 352.5 °C, and the corresponding heat absorption is 28.62 J/g. During the endothermic period, the TG curve of the corresponding sample (PTFE/TiH_2_) is kept parallel to the temperature axis; thus, the mass of the sample remains unchanged. A large exothermic peak B appears on the DSC curve of PTFE/TiH_2_ at 554.6 °C, corresponding to the temperature range of 532.8–571.3 °C, and the heat release is 565.9 J/g. During the exothermic period, the TG curve of PTFE/TiH_2_ decreases sharply, and the corresponding mass fraction increment of the sample is 33.9%, which indicates that peak B is the exothermic reaction peak of PTFE/TiH_2_. It is shown that the exothermic reaction of material Ti and gasification PTFE occurs at the temperature range of 532.8–571.3 °C. A larger endothermic peak C appears near peak B, and the corresponding temperature range is 558.3–619.7 °C; its absorption heat is 1006 J/g. During the endothermic period, the TG curve of PTFE/TiH_2_ material remains stable; thus, the quality of PTFE/TiH_2_ material does not change, indicating that peak C is the melting endothermic peak of PTFE/TiH_2_ material.

**Figure 17:**
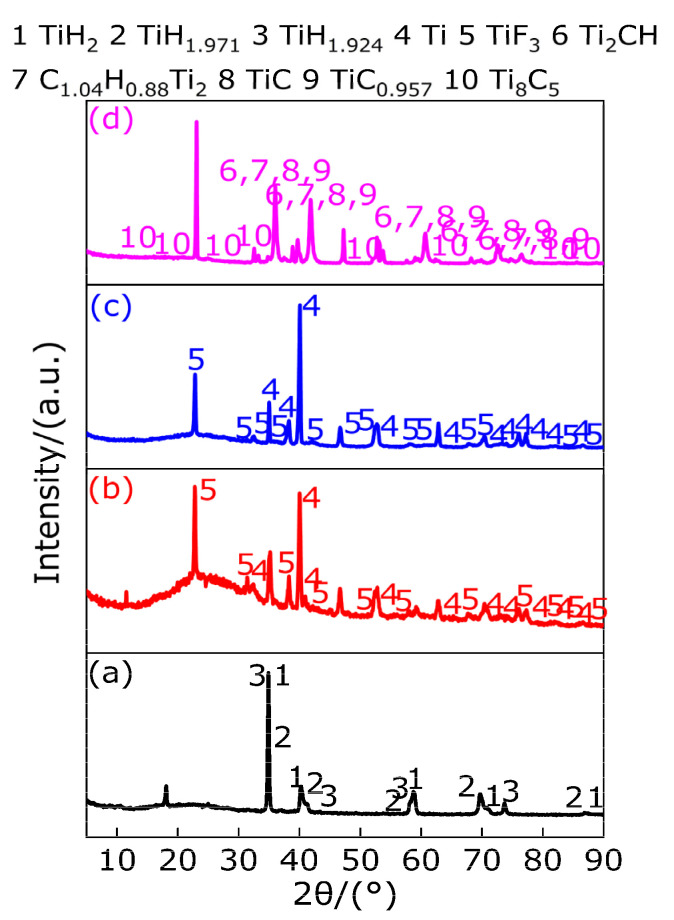
XRD patterns of PTFE/TiH_2_ composite at different peak temperatures and 1000 °C. (**a**) XRD pattern at 340.6 °C; (**b**) XRD pattern at 554.6 °C; (**c**) XRD pattern at 597.1 °C; (**d**) XRD pattern at 1000 °C.

Figure 17 shows the XRD patterns of PTFE/TiH_2_ composite at different peak temperatures and 1000 °C. It can be observed from Figure 17 that there exist three kinds of diffraction peaks of titanium hydride at peak temperature of 340.6 °C, which are TiH_2_, TiH_1.971_ and TiH_1.924_, respectively. Titanium hydride is a nonstoichiometric compound and has a wide variety of species. Generally speaking, compounds with TiH_1.8_-TiH_1.99_ composition are collectively referred to as titanium hydride (TiH_2_). Therefore, there exist three kinds of titanium hydride (TiH_2_, TiH_1.971_ and TiH_1.924_) in the temperature range of 328.5–352.5 °C while PTFE only transforms from the internal crystalline zone to the amorphous gel-alike, which indicates that, in the argon atmosphere, TiH_2_ does not react with PTFE in this temperature range, and TiH_2_ first loses 0.029 hydrogen and converts to TiH_1.971_. With the increase in temperature, TiH_1.971_ lost 0.047 hydrogen again and converted to TiH_1.924_. With the increase in temperature, there exist diffraction peaks of Ti and TiF_3_ at the peak temperature of 554.6 °C of exothermic peak B and at the peak temperature of 597.1 °C of melt absorption peak C, which indicates that in the temperature range of 532.8–619.7 °C, Ti is produced by the dehydrogenation of titanium hydride (TiH_2_, TiH_1.971_ and TiH_1.924_). Part of Ti and gasified PTFE undergo exothermic reaction to produce TiF_3_. It should be noted that there is no decomposition endothermic peak of PTFE on DSC curve of PTFE/TiH_2_ material in this temperature range. The analysis shows that this is caused by the result of the superposition of the exothermic peak of reaction between Ti and PTFE, and the decomposition endothermic peak of PTFE reflects an exothermic peak, namely the endothermic decomposition of PTFE, titanium hydride (TiH_2_, TiH_1.971_ and TiH_1.924_) and exothermic reaction of Ti and PTFE act together. In addition, in this temperature range, the reaction of Ti with PTFE may produce TiF_4_, or the disproportionation reaction of TiF_3_ produces TiF_4_ and metal Ti [42]. There is no diffraction peak of TiF_4_ in the XRD pattern of the residue at the peak temperature of peak B and C, and this is because the boiling point of TiF_4_ is lower and it is easy to gasify and escape [42]. When the temperature increases from 619.7 °C to 1000 °C, the diffraction peaks of Ti_2_CH, C_1.04_H_0.88_Ti_2_, TiC, TiC_0.957_ and Ti_8_C_5_ appear on the XRD patterns, which indicated that at least two of the three elements of Ti, H and C (the product of PTFE above 596.2 °C) reacted to produce the above substances in this temperature range. Combined with the above analysis, the main chemical reactions of PTFE/TiH_2_ composites heated in argon atmosphere are as follows (18)–(31).

TiH_2_ → TiH_1.971_ + 0.029H (18)

TiH_1.971_ → Ti + 1.971H (19)

TiH_1.971_ → TiH_1.924_ + 0.047H (20)

TiH_1.924_ → Ti + 1.924H (21)

H + H → H_2_(g) (22)

(–C_2_F_4_^−^) → C_2_F_4_(g) (23)

4Ti + 3C_2_F_4_ → 4TiF_3_ + 6C (24)

Ti + C_2_F_4_ → TiF_4_(g) + 2C (25)

4TiF_3_ → 3TiF_4_(g) + Ti (26)

C + H + 2Ti → Ti_2_CH (27)

Ti + C → TiC(28)

Ti + 0.957C → TiC_0.957_ (29)

1.04C + 0.88H + 2Ti → C_1.04_H_0.88_Ti_2_ (30)

8Ti + 5C → Ti_8_C_5_ (31)

According to the above analysis, the products of PTFE/TiH_2_ composites in different temperature ranges under argon atmosphere are listed in Table 16.

### 3.6. Pyrolysis Behavior and Reaction Process of Aluminum/Polytetrafluoroethylene (Al/PTFE)

**Figure 18:**
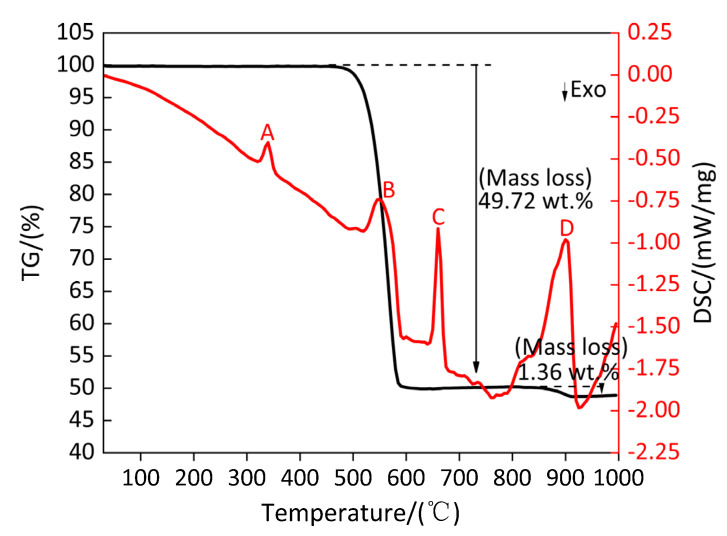
TG/DSC curve of Al/PTFE Composite. A-D: Endothermic peaks.

Figure 18 shows the TG/DSC curve of Al/PTFE composite. Table 17 shows the parameters of peak A, B, C and D on the DSC curve of Al/PTFE composite. It can be observed from Figure 18 and Table 17 that a very small peak A appears on the DSC curve, the corresponding temperature range is 327.2–351.3 °C, and the absorbed heat is 29.69 J/g. During the endothermic period, the TG curve shows that the mass of the sample (Al/PTFE) does not change, indicating that peak A is the melting endothermic peak of the sample (Al/PTFE). With the increase in temperature, an endothermic peak B appears on the DSC curve at 560.8 °C, and the corresponding endothermic starts at 527.5 °C and ends at 587.8 °C, which absorbs heat of 257.4 J/g. During the endothermic period, the mass of the sample (Al/PTFE) decreases sharply, and the mass loss of the sample is 49.72%, which indicates that peak B is the endothermic decomposition peak of the sample (Al/PTFE), and this temperature range is not much different from the temperature range of the decomposition endothermic peak of PTFE in Section 3.1. However, there is no endothermic peak of PTFE decomposition on the DSC curve, which may be caused by the heat generated by the exothermic reaction occurring between Al, and PTFE covers the heat absorbed by PTFE. As the temperature continues to increase, a relatively fine exothermic peak C appears at 660.6 °C, the corresponding temperature range is 650.2–671.8 °C and the absorbed heat is 128.5 J/g. During the endothermic period, the TG curve almost keeps parallel to the temperature axis, indicating that the peak C is the endothermic melting peak of Al/PTFE. At 903.1 °C, a larger endothermic peak D appears on the DSC curve. During the endothermic period, the TG curve corresponding to the temperature range of 857.7–917.9 °C decreases slowly, and the mass loss of the sample (Al/PTFE) is 1.36%, indicating that peak D is the endothermic decomposition peak of the sample (Al/PTFE), which may be due to the mass loss caused by the partial endothermic sublimation of AlF_3_ formed by the reaction of Al with PTFE.

**Figure 19:**
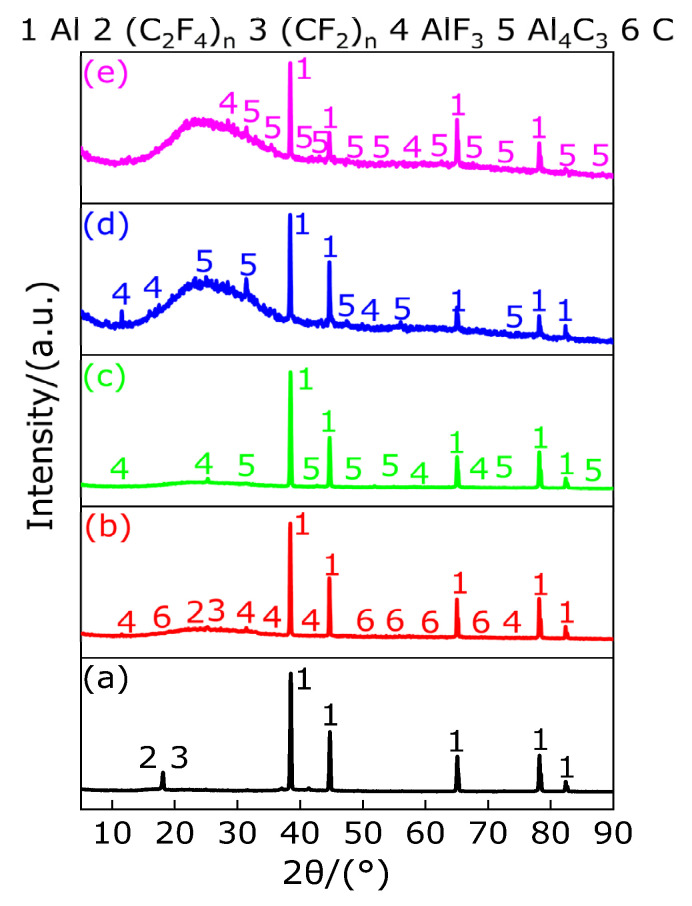
XRD patterns of the residues of Al/PTFE composites at the peak temperatures of peak A, B, C and D on the DSC curve and at 1000 °C. (**a**) XRD pattern at 338.9 °C; (**b**) XRD pattern at 560.8 °C; (**c**) XRD pattern at 660.6 °C; (**d**) XRD pattern at 903.1 °C; (**e**) XRD pattern at 1000 °C.

Figure 19 shows XRD patterns of the residues of Al/PTFE composites at the peak temperatures of peak A, B, C and D on the DSC curve and at 1000 °C. It can be observed from Figure 19 that, at the peak temperature of 338.9 °C of peak A, there exist diffraction peaks of large molecule weight (C_2_F_4_)_n_, small molecule weight (CF_2_)_n_ and Al. It shows that Al does not react with PTFE in the temperature range of 327.2–351.3 °C, but part of the long chain of PTFE breaks into large molecule weight (C_2_F_4_)_n_ and small molecule weight (CF_2_)_n_, which indicates that with the increase in temperature in the temperature range of 327.2–351.3 °C, PTFE transforms from a crystalline and molten state into amorphous PTFE fluid, and the molecules in this fluid are mainly composed of partial PTFE long chain, large molecule weight (C_2_F_4_)_n_ and small molecule weight (CF_2_)_n_. At the peak temperature of 560.8 °C of peak B, XRD patterns show that there exist diffraction peaks of large molecule weight (C_2_F_4_)_n_, small molecule weight (CF_2_)_n_, Al and a new substance AlF_3_. It shows that the endothermic decomposition of PTFE produces gases CF_4_, C_3_F_6_, C_4_F_8_ and C_2_F_6_ in the temperature range of 527.5–587.8 °C, and part of Al reacts with the produced gas and the large molecule weight (C_2_F_4_)_n_ and small molecule weight (CF_2_)_n_ to produce AlF_3_ and carbon black (C) at the same time. At the peak temperature of 660.6 °C of peak C, XRD patterns show that there exist diffraction peaks of Al, AlF_3_ and Al_4_C_3_, indicating that the product C reacts with part of Al to produce Al_4_C_3_ in the temperature range of 650.2–671.8 °C. At the peak temperature of 903.1 °C of peak D, corresponding to the temperature range of 857.7–917.9 °C, XRD diffraction shows that there still exists diffraction peaks of Al, AlF_3_ and Al_4_C_3_. It can also be found from Figure 19 that the intensity of AlF_3_ diffraction peak at 1000 °C is weaker than that at the peak temperature of peak D, the intensity of diffraction peak of AlF_3_ at 1000 °C is weaker than that of AlF_3_ at the peak temperature of peak D and the intensity of diffraction peak of AlF_3_ at peak temperature of peak D is weaker than that of AlF_3_ at the peak temperature of peak C and B, which is caused by the gradual endothermic sublimation of AlF_3_ with the increase in temperature starting from 560.8 °C. Based on the above analysis, the main chemical reactions of Al/PTFE composites during heating in Ar atmosphere are as follows (32)–(40).

(–C_2_F_4_^−^)_n_ → (C_2_F_4_)_n_(g) (32)

(–C_2_F_4_^−^)_n_ → 2(CF_2_)_n_(g) (33)

Al + C_2_F_4_ → 4AlF_3_ + 6C (34)

2Al + 3CF_2_ → 2AlF_3_ + 3C (35)

4Al + 3CF_4_(g) → 4AlF_3_ + 3C (36)

2Al + C_3_F_6_(g) → 2AlF_3_ + 3C (37)

8Al + 3C_4_F_8_(g) → 8AlF_3_ + 12C (38)

2Al + C_2_F_6_(g) → 2AlF_3_ + 2C (39)

4Al + 3C → Al_4_C_3_ (40)

According to the above analysis, the products of Al/PTFE composites in different temperature ranges under argon atmosphere are listed in Table 18.

### 3.7. Pyrolysis Behavior and Reaction Process of Al-rich Al/PTFE/TiH_2_

**Figure 20:**
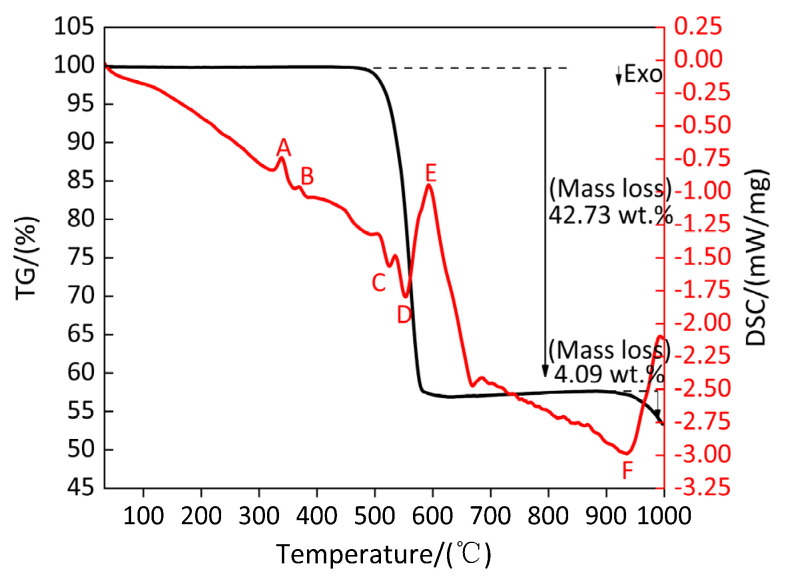
TG/DSC curve of Al-rich Al/PTFE/TiH_2_ composite. A, B and E: Endothermic peak; C, D and F: Exothermic peak.

**Figure 21:**
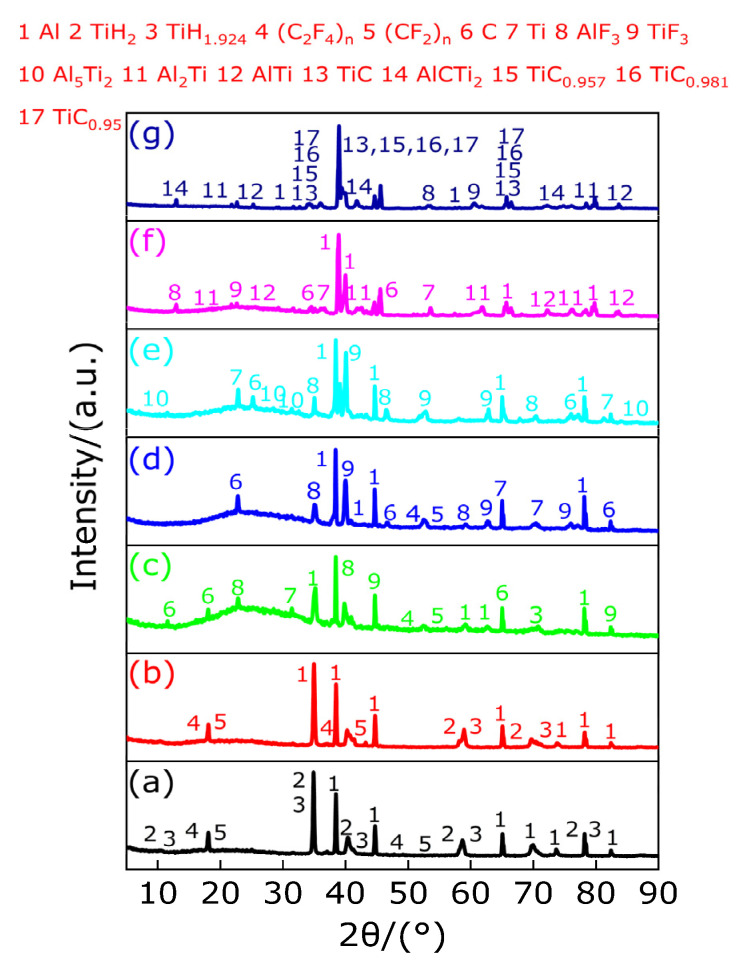
XRD patterns of the residue of the sample (Al-rich Al/PTFE/TiH_2_ composite) at different peak temperatures and 1000 °C. (**a**) XRD pattern at 339.4 °C; (**b**) XRD pattern at 370.1 °C; (**c**) XRD pattern at 523.7 °C; (**d**) XRD pattern at 553.6 °C; (**e**) XRD pattern at 594.5 °C; (**f**) XRD pattern at 942 °C; (**g**) XRD pattern at 1000 °C.

Figure 20 shows the TG/DSC curve of Al-rich Al/PTFE/TiH_2_ composite. Table 19 shows the endothermic/exothermic peak parameters of Al-rich Al/PTFE/TiH_2_ composites. It can be observed from Figure 20 and Table 19 that an endothermic peak A appears on the DSC curve at 339.4 °C and the corresponding temperature range is 328.6–350.6 °C, and the TG curve corresponding to this temperature range is always keep smooth and steady, indicating that peak A is the endothermic melting peak of PTFE in the temperature range of 328.6–350.6 °C. At 370.1 °C, a very small endothermic peak B appears on the DSC curve, and its temperature range is 361.4–378.6 °C. However, the corresponding TG curve remained stable and unchanged. According to Figure 21, the residue at peak B was substance TiH_1.924_; thus, it is known that the endothermic peak B is caused by the removal of 0.076 hydrogen from TiH_2_, but TiH_1.924_ still belongs to the category of TiH_2_, and the corresponding TG curve remained stable and almost unchanged. With the increase in temperature, two small exothermic peaks C and D and a large endothermic peak E appear successively on the DSC curve. It is obvious that the result of joint action of the large endothermic peak E and two small exothermic peaks C and D causes the TG curve to show that the mass of the sample (Al-rich Al/PTFE/TiH_2_ composite) decreases sharply by 42.73%, indicating that in the temperature range of 510.8–565.3 °C, peaks C and D are exothermic decomposition peaks of the sample (Al-rich Al/PTFE/TiH_2_ composite). In the temperature range of 575.5–618.1 °C, peak E is the endothermic decomposition peak of the sample (Al-rich Al/PTFE/TiH_2_ composite). It is considered that this is caused by the joint action of the energy released by endothermic reaction of Al/PTFE and Ti/PTFE and endothermic decomposition of PTFE and TiH_2_. At 942 °C, an exothermic peak F appears on the DSC curve, and the heat release started at 918.5 °C and ended at 963.4 °C. The corresponding TG curve showed that the mass loss of the sample (Al-rich Al/PTFE/TiH_2_ composite) was 4.09%, indicating that, within the temperature range of 918.5–963.4 °C, the peak F is the exothermic reaction peak of the sample (Al-rich Al/PTFE/TiH_2_ composite). It is considered that the mass loss of the sample (Al-rich Al/PTFE/TiH_2_ composite) is caused by the joint effect of AlF_3_ sublimation and TiF_3_ disproportionation reaction.

Figure 21 shows the XRD patterns of the sample (Al-rich Al/PTFE/TiH_2_ composite) residues at different peak temperatures and 1000 °C. It can be observed from Figure 21 that XRD patterns show that there exist diffraction peaks of Al, TiH_2_, TiH_1.924_, (C_2_F_4_)_n_ and (CF_2_)_n_ at the peak temperature of 339.4 °C of peak A and that of 370.1 °C of peak B. This indicates that, in the temperature range of 328.6–378.6 °C, with the increase in temperature, the long chain of partial PTFE breaks into large molecule weight (C_2_F_4_)_n_ and small molecule weight (CF_2_)_n_, and 0.076 hydrogen is removed from TiH_2_ and converted into TiH_1.924_ at the same time. It is indicated that there is no reaction occurring among Al, TiH_2_ and PTFE in the temperature range of 328.6–378.6 °C but only long chain fracture of PTFE and dehydrogenation of TiH_2_. At the peak temperature of 523.7 °C of peak C, XRD patterns show that there exist diffraction peaks of Al, TiH_1.924_, large molecule weight (C_2_F_4_)_n_, small molecule weight (CF_2_)_n_, Ti, AlF_3_ and TiF_3_, indicating that most of TiH_1.924_ is completely dehydrogenated and converted to Ti in the temperature range of 510.8–534.3 °C and, at the same time, AlF_3_, TiF_3_ (or TiF_4_, low boiling point and easy sublimation) and carbon black (C) are produced by the reaction of partial Al and Ti with large molecule weight (C_2_F_4_)_n_ and small molecule weight (CF_2_)_n_, respectively. At the peak temperature of 553.6 °C of peak D and that of 594.5 °C of peak E, XRD patterns show that there exist diffraction peaks of Al, C, large molecule weight (C_2_F_4_)_n_, small molecule weight (CF_2_)_n_, Ti, AlF_3_, TiF_3_ and Al_5_Ti_2_, indicating that in the range of 540.8–618.1 °C, endothermic decomposition of most of PTFE releases gases CF_4_, C_3_F_6_, C_4_F_8_ and C_2_F_6_. Moreover, long chains of a small part of PTFE break into large molecule PTFE ((C_2_F_4_)_n_) and small molecule polytetrafluoroethylene ((CF_2_)_n_), which react with partial Al, and Ti can be generated by dehydrogenation of all TiH_1.924_, respectively, to form AlF_3_, TiF_3_ and carbon black (C), while Al and Ti, which are not fully involved in the reaction, react to form Al_5_Ti_2_. At the peak temperature of 942 °C of peak F, XRD patterns show that there exist diffraction peaks of AlCTi_2_, Al_2_Ti, AlTi, TiC, AlF_3_, Al, TiF_3_, TiC_0.957_, TiC_0.981_ and TiC_0.95_, indicating that at least two elements of Ti, Al and C react in the temperature range of 918.5–1000 °C. In addition, XRD patterns show that AlF_3_ and TiF_3_ decreased gradually from 918.5 °C to 1000 °C, which further proves that exothermic peak F is caused by endothermic sublimation of AlF_3_ and disproportionation endothermic reaction of TiF_3_. Based on the above analysis, the main chemical reactions occurring during the heating process of Al-rich Al/PTFE/TiH_2_ composites are as follows (41)–(60).

(–C_2_F_4_^−^)_n_ → (C_2_F_4_)_n_(g) (41)

(–C_2_F_4_^−^)_n_ → 2(CF_2_)_n_(g) (42)

TiH_2_ → TiH_1.924_ + 0.076H (43)

4Al + 3C_2_F_4_ → 4AlF_3_ + 6C (44)

4Ti + 3C_2_F_4_ → 4TiF_3_ + 6C (45)

3Ti + 3C_2_F_4_ → 3TiF_4_ + 6C (46)

TiH_1.924_ → Ti + 1.924H (47)

H + H → H_2_(g) (48)

4Al + 3CF_4_(g) → 4AlF_3_ + 3C (49)

2Al + C_3_F_6_(g) → 2AlF_3_ + 3C (50)

8Al + 3C_4_F_8_(g) → 8AlF_3_ + 12C (51)

2Al + C_2_F_6_(g) → 2AlF_3_ + 2C (52)

5Al + 2Ti → Al_5_Ti_2_ (53)

Al + C + 2Ti → AlCTi_2_ (54)

2Al + Ti → Al_2_Ti (55)

Al + Ti → AlTi(56)

Ti + C → TiC(57)

Ti + 0.981C → TiC_0.981_ (58)

Ti + 0.957C → TiC_0.957_ (59)

Ti + 0.95C → TiC_0.95_ (60)

According to the above analysis, the products of Al-rich Al/PTFE/TiH_2_ composites in different temperature ranges under argon atmosphere are listed in Table 20.

### 3.8. Combustion Calorific Value Measurement and Chemical Reaction of Active Materials in Oxygen Atmosphere

**Figure 22:**
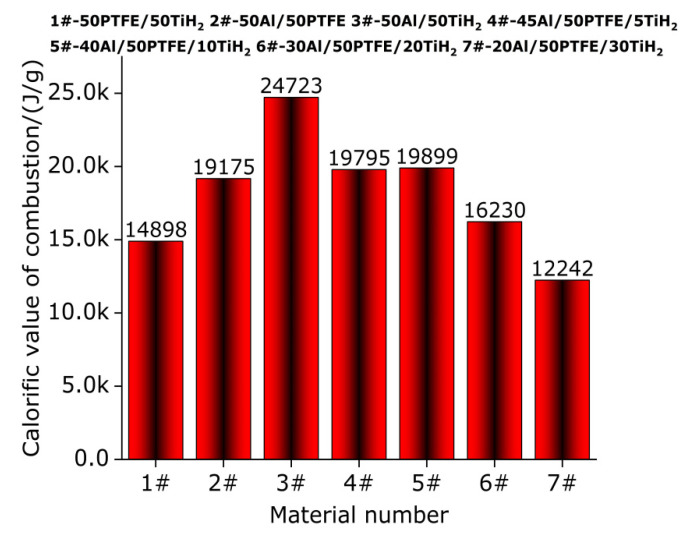
Calorific value of oxygen bomb combustion of active materials.

Column Figure 22 shows the measurement results of combustion calorific value of oxygen bomb of active materials (1#, 2#, 3#, 4#, 5#, 6# and 7#). It can be observed from the experimental data of oxygen bomb in Figure 22 that for the active materials (1#, 2# and 3#) composed of two kinds of materials, the combustion calorific value of 3# is the largest and is 24,723 J/g, which is 28.934% higher than that of 2#. In conclusion, the combustion calorific value of the active materials composed of two kinds of materials with same chemical mass ratio increases gradually. For the active materials (4#, 5#, 6# and 7#) composed of three kinds of materials, the combustion calorific value of 5# is the highest and is 19,899 J/g, which is 62.547% higher than that of 7#. Active material 7# is 0.525% higher than that of 4#, which is 3.776% higher than that of 2#. In conclusion, with the increase in TiH_2_ content, the calorific value of oxygen bomb combustion of active materials composed of three kinds of materials first increased and then decreased. According to the combustion calorific value data of all the active materials in the oxygen bomb experiment, the combustion calorific value of 3# is the largest, which is 24.242% higher than that of 5#. The calorific value of oxygen bomb combustion of 2# is 38.829% higher than that of Al/PTFE with a chemical equilibrium mass ratio of 26.5/73.5 [31], which is 18.731% higher than that of Al/PTFE/TiH_2_ [31] with 30% the content of TiH_2_. Al/PTFE/TiH_2_ [31] with 30% the content of TiH_2_ is 28.899% higher than that of Al/PTFE/TiH_2_ [31] with 10% the content of TiH_2_, which is 23.950% higher than that of Al/PTFE/MoO_3_ [43] with 43% the content of MoO_3_. The calorific value of oxygen bomb combustion of 5# is 33.766% higher than that of Al/PTFE/TiH_2_ [31] with 10% the content of TiH_2_, which is 23.214% higher than that of Al/PTFE/TiH_2_ [31] with 30% the content of TiH_2_, and Al/PTFE/TiH_2_ [31] with 30% the content of TiH_2_ is 28.630% higher than that of Al/PTFE/MoO_3_ [43] with 43% the content of MoO_3_. Based on the above data, the oxygen bomb calorific value of Al/TiH_2_ active material is the highest when TiH_2_ is added to Al, and the oxygen bomb calorific value of Al/PTFE active material is the highest when TiH_2_ is added to Al-rich Al/PTFE active material, that of which is higher than the Al/PTFE active materials with different contents of TiH_2_ and MoO_3_ under the equilibrium state. Therefore, TiH_2_ does play a role in increasing the energy of Al-rich Al/PTFE active materials, which highlights the role of TiH_2_ material as a high-energy additive.

**Figure 23:**
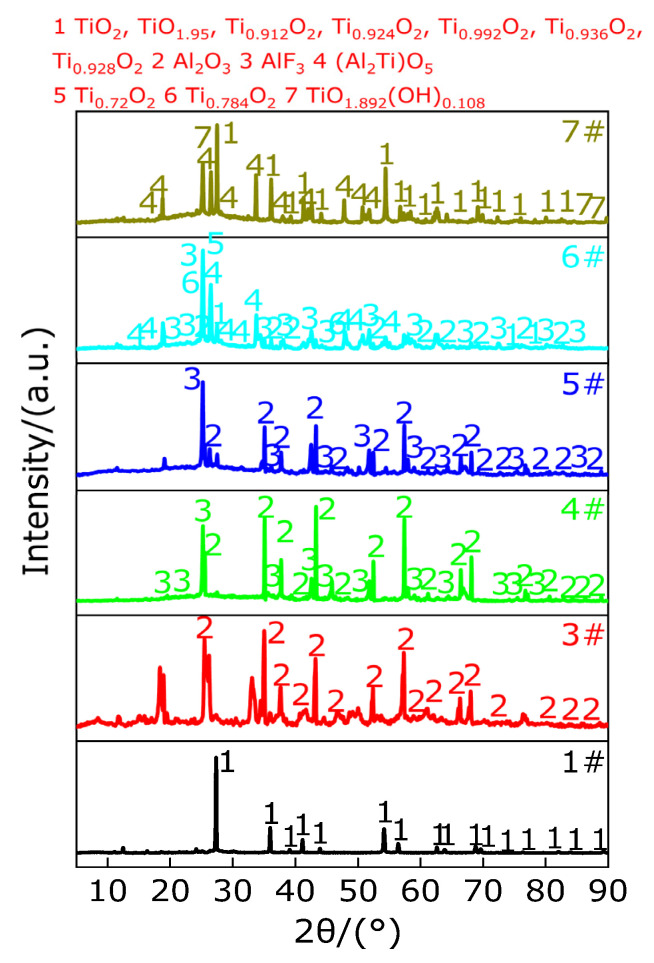
XRD patterns of oxygen bomb experiment residues of materials 1#, 3#, 4#, 5#, 6# and 7#. 1#, 3#, 4#, 5#, 6# and 7#: XRD patterns of oxygen bomb of material 1#, 3#, 4#, 5#, 6# and 7#.

The energy released from the combustion of the material measured by the oxygen bomb experiment is mainly reflected in the sum of the energy released by various reactions of the active material in the oxygen atmosphere. In order to explore the various reactions of active materials in the oxygen atmosphere, only the oxygen bomb experiment residues of active materials 1#, 3#, 4#, 5#, 6# and 7# are collected, and there is no residue after the oxygen bomb experiment of active material 2#. XRD composition analyses of oxygen bomb residues of different numbering materials are carried out. The results are shown in Figure 23. According to the recovered material residue of the oxygen bomb experiment, it can be found that there exist no black matter carbon black, which indicates that carbon black has completely reacted in oxygen atmosphere to form carbon dioxide (CO_2_), and the material is fully burned. It can be observed from Figure 23 that for active material 1# (PTFE/TiH_2_), the XRD patterns show that there exist diffraction peaks of TiO_2_, TiO_1.95_, Ti_0.912_O_2_, Ti_0.924_O_2_, Ti_0.992_O_2_, Ti_0.936_O_2_ and Ti_0.928_O_2_, indicating that PTFE endothermic decomposition releases gases (C_2_F_4_)_n_, (CF_2_)_n_, CF_4_, C_2_F_6_ and carbon black (C) and at the same time different Ti phases and hydrogen (H_2_) are obtained by continuous dehydrogenation of TiH_2_, and the product carbon black, hydrogen and different Ti phases were fully reacted with oxygen to produce CO_2_, water vapor and the above detected substances. The oxygen bomb experiment residue of active material 2# (Al/PTFE) does not exist, which indicates that Al may react with the gases produced by the endothermic decomposition of PTFE to produce AlF_4_ with lower boiling point and carbon black. With the increase in temperature in the oxygen bomb instrument, AlF_4_ sublimates endothermically, and carbon black reacts with oxygen to produce carbon dioxide (CO_2_). For active material 3# (Al/TiH_2_), XRD patterns only show the diffraction peak of Al_2_O_3_ exists, and no oxidation product of pure titanium is detected. It is believed that the reason may be that Ti was produced after the dehydrogenation of TiH_2_, and other metal impurities with mass accounting for a small proportion in Al powder are oxidized by oxygen to produce oxides containing other metal non pure titanium, which indicates that most of the Al_2_O_3_ is mainly produced by the oxidation of Al. At the same time, Ti and H_2_ produced by dehydrogenation of TiH_2_, Ti and other metal impurities are oxidized to produce nonpure titanium oxide, and H_2_ is oxidized to produce water vapor. The energy released by active material 3# is the largest, and it may be caused by the energy released by TiH_2_ in Al being close to the maximum limit. The composition of residue of oxygen bomb experiment of Al-rich Al/PTFE/TiH_2_ active materials with different content of TiH_2_ (4#, 5#, 6# and 7#) is not identical. The composition of residue of oxygen bomb experiment of Al-rich Al/PTFE/TiH_2_ active material with TiH_2_ content of not more than 10% (4# and 5#) is almost the same. XRD patterns show that there exist diffraction peaks of Al_2_O_3_ and AlF_3_, and no Ti related products are detected; this may be caused by the reaction of Ti produced by dehydrogenation of TiH_2_ with PTFE to produce TiF_4_ with lower boiling point and easy sublimation. The main reaction includes the reaction of Al with PTFE to form AlF_3_ and C and Al, and carbon black and hydrogen are fully oxidized to form Al_2_O_3_, CO_2_ and water vapor. Aluminum, carbon black and hydrogen are fully oxidized to form Al_2_O_3_, CO_2_ and water vapor. For active material 6# (Al/PTFE/TiH_2_ with 20% the content of TiH_2_), XRD patterns show that there exist diffraction peaks of TiO_2_, Al_2_O_3_, AlF_3_, (Al_2_Ti)O_5_, Ti_0.72_O_2_ and Ti_0.784_O_2_. Therefore, the main reactions include the reaction of Al with PTFE to produce AlF_3_ and C, dehydrogenation of TiH_2_ to produce Ti and H_2_, oxidation of H_2_ to produce water vapor, partial Al and Ti oxidized to produce (Al_2_Ti)O_5_, oxidation of Ti to produce TiO_2_, oxidation of partially unreacted Al to produce Al_2_O_3_ and partially unreacted Ti with oxygen to produce Ti_0.72_O_2_ and Ti_0.784_O_2_. For material 7# (Al/PTFE/TiH_2_ with 30% the content of TiH_2_), XRD show that there exist diffraction peaks of TiO_2_, TiO_2.95_, Ti_0.912_O_2_, Ti_0.924_O_2_, Ti_0.992_O_2_, Ti_0.936_O_2_, Ti_0.928_O_2_, (Al_2_Ti)O_5_ and TiO_2.892_(OH)_0.108_. Therefore, the main reaction includes the dehydrogenation of TiH_2_ to produce Ti and H_2_. After Ti and H_2_ are oxidized, the above pure titanium oxides and water vapor are produced. No diffraction peaks of AlF_4_ and TiF_4_ are detected. It is believed that this may be caused by the exothermic sublimation of AlF_4_ and TiF_4_. AlF_4_ and TiF_4_ are produced by the reaction of Al and Ti with PTFE, respectively. Partially unreacted Ti is oxidized with Al and H_2_ to form (Al_2_Ti)O_5_ and TiO_1.892_(OH)_0.108_, respectively. Based on the above analysis, the main reactions of active materials under oxygen atmosphere may be as follows.

For 1# (61–74), we have the following.

(–C_2_F_4_^−^)_n_ → (C_2_F_4_)_n_ (61)

(–C_2_F_4_^−^)_n_ → 2(CF_2_)_n_ (62)

(–C_2_F_4_^−^)_n_ → nCF_4_(g) + nC (63)

6(–C_2_F_4_^−^)_n_ → 4nC_2_F_6_(g) + 4nC (64)

C + O_2_ → CO_2_(g) (65)

TiH_2_ → Ti + H_2_(g) (66)

H_2_ + O_2_ → H_2_O(g) (67)

Ti + O_2_ → TiO_2_ (68)

2Ti + 1.95O_2_ → 2TiO_1.95_ (69)

0.912Ti + O_2_ → Ti_0.912_O_2_ (70)

0.924Ti + O_2_ → Ti_0.924_O_2_ (71)

0.992Ti + O_2_ → Ti_0.992_O_2_ (72)

0.936Ti + O_2_ → Ti_0.936_O_2_ (73)

0.928Ti + O_2_ → Ti_0.928_O_2_ (74)

For 2# (75–80), we have the following.

(–C_2_F_4_^−^)_n_ → (C_2_F_4_)_n_(g) (75)

Al + C_2_F_4_ → AlF_4_(g) + C (76)

Al + 2CF_2_ → AlF_4_(g) + 2C (77)

Al + CF_4_ → AlF_4_(g) + C (78)

6Al + 4C_2_F_6_ → 6AlF_4_(g) + 8C (79)

C + O_2_ → CO_2_(g) (80)

For 3# (81–83), we have the following.

TiH_2_ → Ti + H_2_(g) (81)

2H_2_ + O_2_ → 2H_2_O(g) (82)

4Al + 3O_2_ → 2Al_2_O_3_ (83)

For 4# and 5#, (84–90), we have the following.

(–C_2_F_4_^−^)_n_ → (C_2_F_4_)_n_(g) (84)

TiH_2_ → Ti + H_2_(g) (85)

H_2_ + O_2_ → H_2_O(g) (86)

4Al + 4C_2_F_4_ → 4AlF_3_ + 6C (87)

Ti + C_2_F_4_ → TiF_4_(g) + 2C (88)

C + O_2_ → CO_2_(g) (89)

Al + O_2_ → Al_2_O_3_ (90)

For 6# (91–100), we have the following.

(–C_2_F_4_^−^)_n_ → (C_2_F_4_)_n_(g) (91)

TiH_2_ → Ti + H_2_(g) (92)

2H_2_ + O_2_ → 2H_2_O(g) (93)

4Al + 3C_2_F_4_ → 4AlF_3_ + 6C (94)

C + O_2_ → CO_2_(g) (95)

Ti + O_2_ → TiO_2_ (96)

Ti + O_2_ → Ti_0.72_O_2_ (97)

Ti + O_2_ → Ti_0.784_O_2_ (98)

Al + O_2_ → Al_2_O_3_ (99)

4Al + 2Ti + 5O_2_ → 2(Al_2_Ti)O_5_ (100)

For 7# (101–115), we have the following.

(–C_2_F_4_^−^)_n_ → (C_2_F_4_)_n_(g) (101)

TiH_2_ → Ti + H_2_(g) (102)

Ti + O_2_ + 0.054H_2_ → TiO_1.892_(OH)_0.108_ (103)

2H_2_ + O_2_ → 2H_2_O(g) (104)

Al + C_2_F_4_ → AlF_4_(g) + 2C (105)

Ti + C_2_F_4_ → TiF_4_(g) + 2C (106)

C + O_2_ → CO_2_(g) (107)

Ti + O_2_ → TiO_2_ (108)

2Ti + 1.95O_2_ → 2TiO_1.95_ (109)

0.912Ti + O_2_ → Ti_0.912_O_2_ (110)

0.924Ti + O_2_ → Ti_0.924_O_2_ (111)

0.992Ti + O_2_ → Ti_0.992_O_2_ (112)

0.936Ti + O_2_ → Ti_0.936_O_2_ (113)

0.928Ti + O_2_ → Ti_0.928_O_2_ (114)

4Al + 2Ti + 5O_2_ → 2(Al_2_Ti)O_5_ (115)

## 4. Conclusions

In order to study the reaction mechanism of active materials in argon and oxygen atmosphere. In this paper, the thermal decomposition and thermal reaction behaviors of the active materials were studied by thermogravimetry differential scanning calorimetry (TG-DSC), and the compositions of TG-DSC residues at different peak temperatures and 1000 °C and residues of oxygen bomb experiment are analyzed by X-ray diffraction (XRD) so as to explore the reaction mechanism of the material in argon and oxygen atmosphere. The main conclusions drawn are as follows:

1. In argon atmosphere, the pyrolysis of PTFE is an endothermic reaction, and the whole pyrolysis process has only one weightlessness stage, which indicates that the pyrolysis process of PTFE is a one-step reaction. When the temperature is lower than 527 °C, the long chain of PTFE breaks into large molecular weight (C_2_F_4_)_n_ and low molecular weight (CF_2_)_n_. When the temperature is in the range of 527–596.2 °C, the products of PTFE have large molecular weight (C_2_F_4_)_n_, low molecular weight (CF_2_)_n_, C_3_F_6_(g), C_4_F_8_(g), CF_4_(g) and C_2_F_6_(g). When the temperature is higher than 596.2 °C, the final product of PTFE decomposition is carbon black (C).
2. A small endothermic decomposition peak and a larger endothermic decomposition peak appear on the DSC curve of TiH_2_ under argon atmosphere. The TG curve shows that the total mass loss of sample (TiH_2_) is 2.97%. Therefore, the pyrolysis of TiH_2_ is a multistage reaction rather than a one-step reaction. When the temperature is in the range of 386.33–442.9 °C, for the first time, 0.5 hydrogen (H) is removed from TiH_2_ and converted to TiH_1.5_, and TiH_1.5_ is a stable existence in the temperature range of 386.33–470.33 °C. When the temperature is in the range of 470.33–523.8 °C, 1.5 hydrogen is removed from partial TiH_1.5_ to produce Ti, which coexists with incomplete decomposed TiH_1.5_. When the temperature is in the range of 650.33–1000 °C, only matter Ti exists.
3. In an argon atmosphere, when the temperature is heated from room temperature to 1000 °C, a melting endothermic peak appears on the DSC curve of Al, and the TG curve shows that the mass of the sample (Al) does not change. Therefore, when the temperature is heated from room temperature to 1000 °C, Al only experiences changes in the physical state without chemical changes in matter, namely, solid state → solid–liquid mixed state → liquid state.
4. The pyrolysis of Al/TiH_2_ samples under argon atmosphere is divided into five stages. In the first stage (below 386.33 °C), Al and TiH_2_ coexist and do not react. In the second stage (386.33–465.1 °C), 0.5 hydrogen (H) is removed from TiH_2_ for the first time and transformed into TiH_1.5_, which coexisted with Al. In the third stage (491.3–545.8 °C), 1.5 hydrogen is removed from TiH_1.5_ to produce Ti, which coexists with Al. In the fourth stage (589.4–671.2 °C), Al reacts with Ti to form Al_3_Ti, which coexists with partial unreaction of Al and Ti. In the fifth stage (671.2–1000 °C), partial unreaction Al reacts with Ti to form Al_2_Ti or AlTi, and the disproportionation reaction of Al_3_Ti occurs to produce Al_2_Ti and AlTi.
5. The pyrolysis of TiH_2_/PTFE under argon atmosphere can be divided into three stages. In the first stage (328.5–352.5 °C), 0.029 hydrogen (H) was removed from TiH_2_ and transformed into TiH_1.971_, then 0.047 hydrogen (H) was removed from TiH_1.971_ and transformed into TiH_1.924_, which coexisted with the undecomposed TiH_2_. In the second stage (532.8–619.7 °C), the endothermic decomposition of PTFE produced C_2_F_4_(g), CF_4_(g), CF_2_(g), C_3_F_6_(g), C_4_F_8_(g), C_2_F_6_(g) and carbon black (C), while 1.924 hydrogen was completely removed from TiH_1.924_ and converted into Ti, which reacted with the above gases to produce TiF_3_ and carbon black (C). In the third stage (619.7–1000 °C), TiC, Ti_2_CH, C_1.04_H_0.88_Ti_2_, TiC_0.957_ and Ti_8_C_5_ are produced by the mutual reaction of at least two of Ti, C and H.
6. The pyrolysis of Al/PTFE under argon atmosphere can be divided into three stages. In the first stage (327.2–351.3 °C), the long chain of partial PTFE breaks into polytetrafluoroethylene with large molecular weight ((C_2_F_4_)_n_) and polytetrafluoroethylene with low molecular weight ((CF_2_)_n_), which coexists with Al. In the second stage (527.5–587.8 °C), PTFE decomposes endothermically to produce gases CF_4_, C_3_F_6_, C_4_F_8_, C_2_F_6_ and carbon black (C), and partial Al reacts with the above gases to generate AlF_3_ and carbon black (C). In the third stage (650.2–1000 °C), Al reacts with C to form Al_4_C_3_, which coexists with partially unreacted Al and AlF_3_. In addition, it can be observed from the XRD pattern that AlF_3_ decreases gradually in the temperature range of 650.2–1000 °C.
7. The pyrolysis of Al-rich Al/PTFE/TiH_2_ under argon atmosphere can be divided into four stages. In the first stage (328.6–378.6 °C), the long chain fracture of PTFE and dehydrogenation of TiH_2_ produced TiH_1.924_, (C_2_F_4_)_n_, (CF_2_)_n_ and H_2_(g), which coexist with partially unreacted Al and TiH_2_. In the second stage (510.8–534.3 °C), 0.076 hydrogen is removed from partially unreacted TiH_2_ and converted to TiH_1.924_, and then 1.924 hydrogen is removed from TiH_1.924_ and converted into Ti. Part of Al and Ti reacts with a part of large and small molecular weight polytetrafluoroethylene to form AlF_3_, TiF_3_ and TiF_4_(g). The main products are Al, TiH_1.924_, (C_2_F_4_)_n_, (CF_2_)_n_, Ti, AlF_3_, TiF_3_, TiF_4_, C and H_2_(g). In the third stage (540.8–618.1 °C), in addition to polytetrafluoroethylene containing large and small molecular weight, PTFE decomposes endothermically to produce gases CF_4_, C_3_F_6_, C_4_F_8_ and C_2_F_6_. Al and Ti react with the above gases and polytetrafluoroethylene with molecular weight to produce AlF_3_, TiF_3_, TiF_4_ and carbon black (C). Partial Al reacts with Ti to produce Al_5_Ti_2_. The main products are Al, C, Ti, (C_2_F_4_)_n_, (CF_2_)_n_, AlF_3_, TiF_3_, TiF_4_, CF_4_(g), C_3_F_6_(g), C_4_F_8_(g), C_2_F_6_(g), Al_5_Ti_2_ and H_2_(g). In the fourth stage (918.5–1000 °C), at least two elements of Ti, Al and C react with one another to form AlCTi_2_, Al_2_Ti, AlTi, TiC, AlF_3_, Al, TiF_3_, TiC_0.957_, TiC_0.981_ and TiC_0.95_.
8. In composite materials containing two kinds of components, the calorific value of oxygen bomb of Al/TiH_2_ composite is the largest and is 24,723 J/g, that of which is 28.934% higher than that of Al-rich Al/PTFE. In composite materials containing three kinds of components, the calorific value of oxygen bomb of Al-rich Al/PTFE/TiH_2_ composite with 10% the content of TiH_2_ is the largest and is 19,899 J/g. The calorific value of the composites composed of two materials with the same chemical mass ratio increases gradually. With the increase in TiH_2_ content, the combustion calorific value of oxygen bomb of Al-rich Al/PTFE/TiH_2_ material first increases and then decreases. Therefore, TiH_2_ does play a role in increasing the energy of Al-rich Al/PTFE active material, which highlights the role of TiH_2_ material as a high-energy additive.
9. In oxygen atmosphere, the reaction mechanism of various materials is different. For PTFE/TiH_2_ material, the main products are as follows: (C_2_F_4_)_n_, (CF_2_)_n_, CF_4_(g), C_2_F_6_(g), CO_2_(g), H_2_(g), C, H_2_O(g), TiO_2_, Ti_0.912_O_2_, Ti_0.924_O_2_, Ti_0.992_O_2_, Ti_0.936_O_2_ and Ti_0.928_O_2_. For the Al/PTFE material, the main products are as follows: C_2_F_4_(g), AlF_4_(g) and CO_2_(g). For Al/TiH_2_ material, the main products are as follows: H_2_(g), H_2_O(g) and Al_2_O_3_. For materials Al-rich Al/PTFE/TiH_2_-5wt% and Al-rich Al/PTFE/TiH_2_-10wt%, the two materials have the same products as follows: C_2_F_4_(g), H_2_(g), H_2_O(g), AlF_3_, TiF_4_(g), CO_2_(g) and Al_2_O_3_. For Al-rich Al/PTFE/TiH_2_-20wt%, the main products are as follows: (C_2_F_4_)_n_(g), H_2_O(g), AlF_3_, CO_2_(g), TiO_2_, Ti_0.72_O_2_, Ti_0.784_O_2_ and Al_2_O_3_. For Al-rich Al/PTFE/TiH_2_-30wt%, the main products are as follows: (C_2_F_4_)_n_(g), TiO_1.892_(OH)_0.108_, H_2_O(g), AlF_4_(g), TiF_4_(g), CO_2_(g), TiO_2_, TiO_1.95_, Ti_0.912_O_2_, Ti_0.924_O_2_, Ti_0.992_O_2_, Ti_0.936_O_2_, Ti_0.928_O_2_ and (Al_2_Ti)O_5_.

## Figures and Tables

**Table 1:** Parameters of experimental raw materials.

Main Raw Material Powder	Average Particle Size/(μm)	Purity/(%)	Production Unit
(Polytetrafluoroethylene)PTFE	27	>99.5	HS, Guangdong, China
Al	6–7	>99.5	AG, Liaoning, China
TiH_2_	4–6	>99.5	RF, Hunan, China
Anhydrous ethanol	/	95	TG, Beijing, China

**Table 2:** The physical and chemical properties of raw materials.

Component	Density/(g/cm^3^)	Appearance	Melting/Boiling Point(K)	Decomposition Point/(K)	Theoretical ∆H of Reaction with Al/(kJ/g)
PTFE	2.200	White powder	614/-	829	28.9
Al	2.702	Silver grey powder	933/2600	-	-
TiH_2_	3.910	Dark grey powder	673/-	973	-

**Table 3:** Material composition ratio of TG/DSC experiment.

NO./Sample	Composition(wt%)		
	Al	PTFE	TiH_2_
1/(PTFE)	/	100	/
2/(TiH_2_)	/	/	100
3/(Al)	100	/	/
4/(Al/TiH_2_)	50	/	50
5/(PTFE/TiH_2_)	/	50	50
6/(Al/PTFE/TiH_2_)	50	50	/
7/(Al/PTFE/TiH_2_)	20	50	30

**Table 4:** Material composition ratio of oxygen bomb test.

NO./Sample	Composition(wt%)		
	Al	PTFE	TiH_2_
1#/(PTFE/TiH_2_)	/	50	50
2#/(Al/PTFE)	50	50	/
3#/(Al/TiH_2_)	50	/	50
4#/(Al/PTFE/TiH_2_)	45	50	5
5#/(Al/PTFE/TiH_2_)	40	50	10
6#/(Al/PTFE/TiH_2_)	30	50	20
7#/(Al/PTFE/TiH_2_)	20	50	30

**Table 5:** Parameters of peak A and peak B.

Name of Peak	Initial Temperature/(°C)	Peak Temperature/(°C)	Termination Temperature/(°C)	Enthalpy of Reaction/(J/g)
Endothermic peak A	330.9	342.2	347.7	63.07
Endothermic peak B	527.0	560.1	596.2	1265

**Table 6:** Pyrolysis products of PTFE in different temperature ranges.

Temperature/(°C)	<527	527–596.2 [34]	>596.2
Decomposition products of PTFE	(CF_2_)_n_, PTFE, (C_2_F_4_)_n_	(C_2_F_4_)_n_, C_3_F_6_(_g_), C_4_F_8_(_g_), CF_4_(g), C_2_F_6_(g), (CF_2_)_n_	C

**Table 7:** Parameters of peak A and peak B on DSC curve of TiH_2_.

Name of Peak	Initial Temperature/(°C)	Peak Temperature/(°C)	Termination Temperature/(°C)	Enthalpy of Reaction/(J/g)
Endothermic peak A	386.33	442.9	470.33	109.2
Endothermic peak B	470.33	523.8	650.33	651.7

**Table 8:** Products of TiH_2_ under argon atmosphere in different temperature ranges.

Temperature/(°C)	<386.33	386.33–650.33	>650.33
Decomposition products of TiH_2_	TiH_2_	TiH_1.5_, Ti	Ti

**Table 9:** Parameters of three stages of thermal decomposition of TiH_2_.

	First Stage	Second Stage	Third Stage
Temperature/(°C)	86.33–386.33	386.33–470.33	470.33–650.33
Initial mass m_1_/(mg)	16.6158	16.5955	16.5002
Termination mass m_2_/(mg)	16.5955	16.5002	16.1223
Loss mass Δm/(mg)	0.0203	0.0953	0.3779
Mass loss time/(min)	59.16362	16.7892	36.05416

**Table 10:** Reaction enthalpy parameters of Ti and H.

Element	Enthalpy of Decomposition Reaction/(kJ/mol)
Ti	$\Delta_{r}G_{m}^{0}\left(298.15K\right)=-80.33$
H	$\Delta_{r}H_{m}^{0}\left(298.15K\right)=-119.66$

**Table 11:** Heat capacity parameters of Ti, H_2_ and TiH_2_.

Substance	Heat Capacity C_m_/(J/(mol•K))
Ti	25.02
H_2_	26.88 + 4.347 × 10^−3^T − 0.3265 × 10^−6^T^2^ + 6.656 × 10^−9^T^3^
TiH_2_	30.12

**Table 12:** Parameters of peak A on DSC curve of sample Al.

Name of Peak	Initial Temperature/(°C)	Peak Temperature/(°C)	Termination Temperature/(°C)	Enthalpy of Reaction/(J/g)
Melting endothermic peak A	654.3	661.7	670.2	299.6

**Table 13:** Parameters of peak A, peak B and peak C on DSC curve of Al/TiH_2_.

Name of Peak	Initial Temperature/(°C)	Peak Temperature/(°C)	Termination Temperature/(°C)	Enthalpy of Reaction/(J/g)
Endothermic peak A	418.9	444.7	465.1	55.64
Endothermic peak B	491.3	523.9	545.8	461.3
Exothermic peak C	598.4	633.8	671.2	−389.7

**Table 14:** Products of samples (Al/TiH_2_) at different temperatures.

Temperature/(°C)	<386.33	386.33–465.1	491.3–545.8	598.4–671.2	671.2–1000
Products of the sample (Al/TiH_2_)	TiH_2_, Al	TiH_1.5_, Al, H_2_(g)	Al, Ti, H_2_(g)	Al, Ti, Al_3_Ti	Al_2_Ti, AlTi

**Table 15:** Parameters of peak A, B and C on DSC curve of sample (PTFE/TiH_2_).

Name of Peak	Initial Temperature/(°C)	Peak Temperature/(°C)	Termination Temperature/(°C)	Enthalpy of Reaction/(J/g)
Endothermic peak A	328.5	340.6	352.5	28.62
Exothermic peak B	532.8	554.6	571.3	−565.9
Endothermic peak C	558.3	597.1	619.7	1006

**Table 16:** Products of PTFE/TiH_2_ composites in different temperature ranges under argon atmosphere.

Temperature/(°C)	328.5–352.5	532.8–619.7	619.7–1000
Products of sample (PTFE/TiH_2_)	TiH_2_, TiH_1.971_, TiH_1.924_, H_2_(g)	Ti, TiF_3_, C, H_2_(g), C_2_F_4_(g), CF_2_(g)	Ti_2_CH, C_1.04_H_0.88_Ti_2_, TiC, TiC_0.957_, Ti_8_C_5_

**Table 17:** Parameters of peak A, B, C and D on DSC curve of Al/PTFE Composite.

Name of Peak	Initial Temperature/(°C)	Peak Temperature/(°C)	Termination Temperature/(°C)	Enthalpy of Reaction/(J/g)
Endothermic peak A	327.2	338.9	351.3	29.69
Endothermic peak B	527.5	560.8	587.8	257.4
Endothermic peak C	650.2	660.6	671.8	128.5
Endothermic peak D	857.7	903.1	917.9	464.3

**Table 18:** Products of Al/PTFE composites in different temperature ranges under argon atmosphere.

Temperature/(°C)	327.2–351.3	527.5–587.8	650.2–1000
Products of sample (PTFE/Al)	Al, (C_2_F_4_)_n_, (CF_2_)_n_	Al, (C_2_F_4_)_n_, (CF_2_)_n_, CF_4_(g), C_3_F_6_(g), C_4_F_8_(g), C_2_F_6_(g), C, AlF_3_	Al, AlF_3_, Al_4_C_3_

**Table 19:** The endothermic/exothermic peak parameters of Al-rich Al/PTFE/TiH_2_ composites.

Name of Peak	Initial Temperature/(°C)	Peak Temperature/(°C)	Termination Temperature/(°C)	Enthalpy of Reaction/(J/g)
Endothermic peak A	328.6	339.4	350.6	29.55
Endothermic peak B	361.4	370.1	378.6	4.615
Exothermic peak C	510.8	523.7	534.3	−22.69
Exothermic peak D	540.8	553.6	565.3	−60.97
Endothermic peak E	575.5	594.5	618.1	264
Exothermic peak F	918.5	942	963.4	−140

**Table 20:** The products of Al-rich Al/PTFE/TiH_2_ composites in different temperature ranges under argon atmosphere.

Temperature/(°C)	328.6–378.6	510.8–534.3	540.8–618.1	918.5–1000
Products of the sample (Al-rich PTFE/Al/TiH_2_)	Al, TiH_2_, TiH_1.924_, (C_2_F_4_)_n_, (CF_2_)_n_, H_2_(g)	Al, TiH_1.924_, (C_2_F_4_)_n_, (CF_2_)_n_, Ti, AlF_3_, TiF_3_, TiF_4_, C, H_2_(g)	Al, C, Ti, (C_2_F_4_)_n_, (CF_2_)_n_, AlF_3_, TiF_3_, TiF_4_, CF_4_(g), C_3_F_6_(g), C_4_F_8_(g), C_2_F_6_(g), Al_5_Ti_2_, H_2_(g)	AlCTi_2_, Al_2_Ti, AlTi, TiC, AlF_3_, Al, TiF_3_, TiC_0.957_, TiC_0.981_, TiC_0.95_
